# Preliminary model assessing the cost-effectiveness of preoperative chlorhexidine mouthwash at reducing postoperative pneumonia among abdominal surgery patients in South Africa

**DOI:** 10.1371/journal.pone.0254698

**Published:** 2021-08-12

**Authors:** Mwayi Kachapila, Adesoji O. Ademuyiwa, Bruce M. Biccard, Dhruva N. Ghosh, James Glasbey, Mark Monahan, Rachel Moore, Dion G. Morton, Raymond Oppong, Rupert Pearse, Tracy E. Roberts, NIHR Global Health Research Unit on Global Surgery, ASOS Investigators, STARSurg Collaborative

**Affiliations:** 1 National Institute for Health Research Global Health Research Unit on Global Surgery, Institute of Translational Medicine, University of Birmingham, Birmingham, England, United Kingdom; 2 Health Economics Unit, Institute of Applied Health Research, College of Medical and Dental Sciences, University of Birmingham, Birmingham, England, United Kingdom; 3 Paediatric Surgery Unit, Department of Surgery, Faculty of Clinical Sciences, College of Medicine, University of Lagos, Lagos, Nigeria; 4 Department of Anaesthesia and Perioperative Medicine, Groote Schuur Hospital and University of Cape Town, Cape Town, South Africa; 5 India Hub National Institute for Health Research Global Health Research Unit on Global Surgery, Ludhiana, India; 6 Surgery Christian Medical College, Ludhiana, India; 7 Department of Surgery, University of the Witwatersrand, Johannesburg, South Africa; 8 Birmingham Surgical Trials Consortium, Institute of Applied Health Research, College of Medical and Dental Sciences, University of Birmingham, Birmingham, England, United Kingdom; 9 Barts and The London School of Medicine and Dentistry, Queen Mary University of London, London, England, United Kingdom; University of Health Sciences, Sisli Hamidiye Etfal Training and Research Hospital, TURKEY

## Abstract

**Background:**

Pneumonia is a common and severe complication of abdominal surgery, it is associated with increased length of hospital stay, healthcare costs, and mortality. Further, pulmonary complication rates have risen during the SARS-CoV-2 pandemic. This study explored the potential cost-effectiveness of administering preoperative chlorhexidine mouthwash versus no-mouthwash at reducing postoperative pneumonia among abdominal surgery patients.

**Methods:**

A decision analytic model taking the South African healthcare provider perspective was constructed to compare costs and benefits of mouthwash versus no-mouthwash-surgery at 30 days after abdominal surgery. We assumed two scenarios: (i) the absence of COVID-19; (ii) the presence of COVID-19. Input parameters were collected from published literature including prospective cohort studies and expert opinion. Effectiveness was measured as proportion of pneumonia patients. Deterministic and probabilistic sensitivity analyses were performed to assess the impact of parameter uncertainties. The results of the probabilistic sensitivity analysis were presented using cost-effectiveness planes and cost-effectiveness acceptability curves.

**Results:**

In the absence of COVID-19, mouthwash had lower average costs compared to no-mouthwash-surgery, $3,675 (R 63,770) versus $3,958 (R 68,683), and lower proportion of pneumonia patients, 0.029 versus 0.042 (dominance of mouthwash intervention). In the presence of COVID-19, the increase in pneumonia rate due to COVID-19, made mouthwash more dominant as it was more beneficial to reduce pneumonia patients through administering mouthwash. The cost-effectiveness acceptability curves shown that mouthwash surgery is likely to be cost-effective between $0 (R0) and $15,000 (R 260,220) willingness to pay thresholds.

**Conclusions:**

Both the absence and presence of SARS-CoV-2, mouthwash is likely to be cost saving intervention for reducing pneumonia after abdominal surgery. However, the available evidence for the effectiveness of mouthwash was extrapolated from cardiac surgery; there is now an urgent need for a robust clinical trial on the intervention on non-cardiac surgery.

## Introduction

Pneumonia is a common and severe complication that occurs after abdominal surgery with incidence rates reported between 4% and 17.5% [1, 2]. Postoperative pneumonia is associated with increased length of hospital stay (LoS), cost of care, morbidity and mortality rates [3]. Prospective cohort data have suggested that pulmonary complications rates are 2 to 3 times higher in low-and middle-income countries (LMIC) than in high-income countries (HICs) [1, 4].

In March 2020, the WHO declared the spread of corona-virus disease (COVID-19) a global pandemic [5, 6]. Research has demonstrated the severe phenotype of SARS-CoV-2 infection in the perioperative setting with 60% of patients suffering pneumonia or other serious pulmonary complication [7]. Whilst the absolute rate of cross-infection is low (2.1% to 3.6%), the global burden of postoperative pneumonia is likely to increase [8]. Preoperative mouthwash decolonises bacteria in the lower respiratory tract thereby blocking the entry point of pneumonia and evidence from cardiac surgery patients has shown that it reduces the risk of postoperative pneumonia [9, 10]. It is plausible that preoperative mouthwash would have a similar effect in other major non-cardiac surgeries, however, there is no evidence from randomised trials to date.

We aimed to assess the potential cost-effectiveness of preoperative chlorhexidine mouthwash when compared to no-mouthwash at reducing postoperative pneumonia among abdominal surgery patients and explore the impact of COVID-19 on the cost-effectiveness results in a LMIC setting (South Africa).

## Methods

We developed a decision analytic model in Microsoft Excel 2016 (Microsoft Corporation, Redmond, Washington, USA). The choice of the model was appropriate due to the short timeframe of the condition [11]. We assessed the costs and benefits of preoperative mouthwash at reducing postoperative pneumonia among abdominal surgery patients compared to no-mouthwash-surgery.

### Model

**Fig 1:**
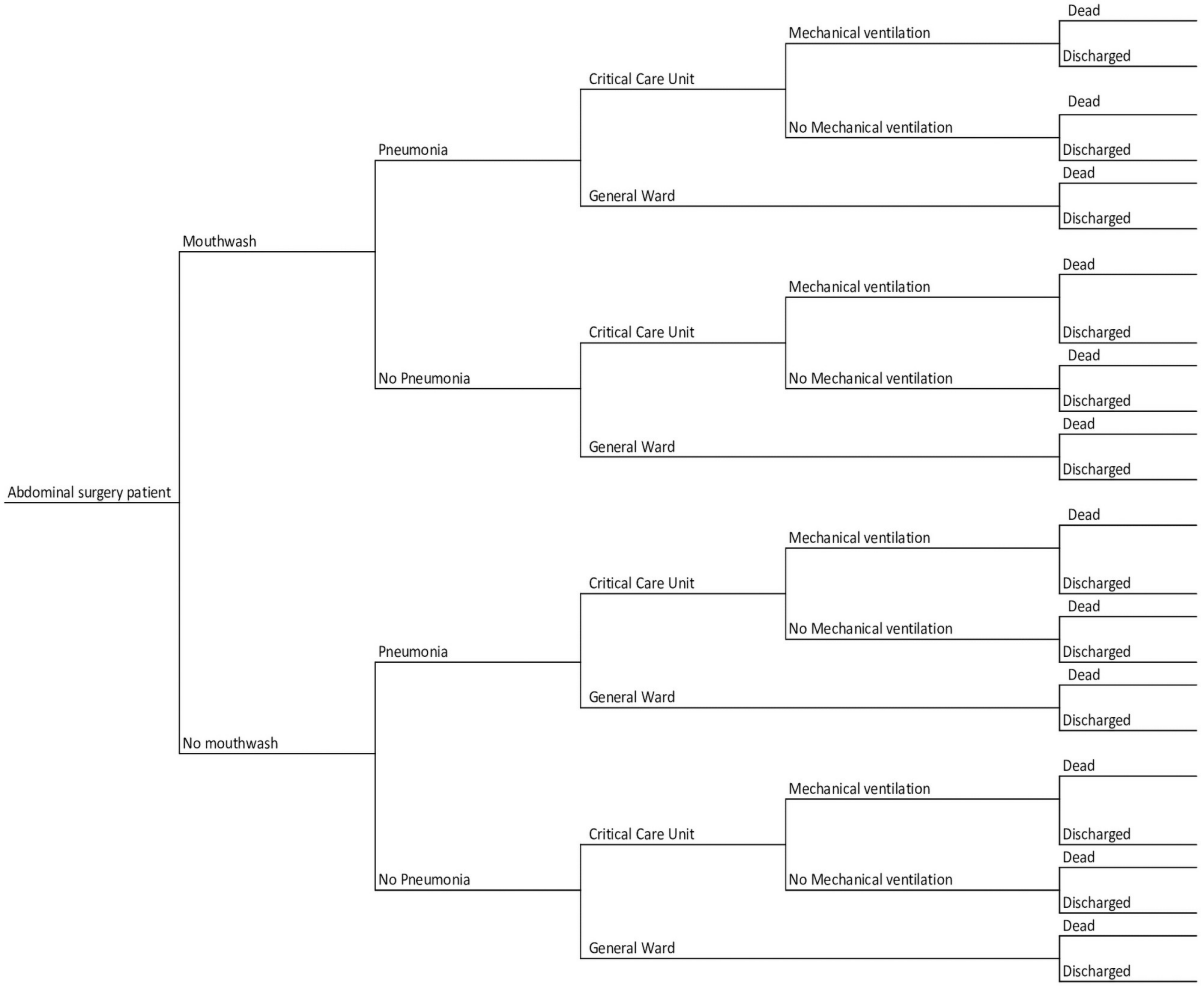
Abdominal surgery patient pathways.

The model structure was developed in consultation with clinical experts from the NIHR Global Health Research Unit on Global Surgery [12]. The agreed model structure, presents identical pathways for mouthwash and no-mouthwash-surgery as well as pneumonia and no pneumonia patients up to the point of discharge. After surgery, a proportion of patients had postoperative pneumonia and were then admitted to either general ward or critical care unit. Further, a proportion of patients admitted to critical care unit needed mechanical ventilation. The critical care unit patients could be discharged home, transferred to a general ward or die by 30 days after surgery. Similarly, patients admitted to general ward, could be discharged home, transferred to critical care unit or die (see Fig 1).

### Model assumptions

The following assumptions were necessary in order to carry out the analysis. Patients in the mouthwash arm were administered chlorhexidine two times immediately before anaesthesia. Mouthwash was more effective than no-mouthwash-surgery at reducing postoperative pneumonia based on data extrapolated from cardiac surgery and mechanical ventilated patients [10, 13]. The expected outcomes of patients who did not develop pneumonia in the experimental and control arms were assumed identical such that the two arms were assigned identical critical care unit, mechanical ventilation and death probabilities. The expected mortality rates for patients who developed pneumonia then admitted to general ward were assumed identical in the intervention and control arms. Patients could be discharged from critical care unit to the general ward and conversely from general ward to critical care unit. We assumed that the time spent in critical care unit by patients who were eventually transferred to general ward was equal to the time spent in general ward by patients who were eventually transferred to critical care unit.

### Model parameters

#### Probabilities

There were no randomised control trials on the effectiveness of mouthwash at reducing pneumonia after abdominal surgery. We therefore derived probability estimates directly from two international prospective cohort studies. Firstly, data for South African patients recruited in the “African Surgical Outcomes Study” (ASOS) were used to estimate most probabilities in the control arm [1]. Secondly, data for the probability of death in general ward among patients with pneumonia and LoS for all the pathways were estimated from the “Respiratory Complications After Abdominal Surgery” (RECON/STARSurg) study database [14]. For three parameters where data were not available, we solicited expert opinion from four anaesthetists and critical care doctors working in South Africa through a survey (see questionnaire in S1 Appendix).

Due of lack of evidence on the intervention among non-cardiac surgery patients, to estimate the probabilities in the intervention arm we relied on evidence that mouthwash reduces pneumonia incidence rate by 0.52 (CI 0.39–0.71) relative risk reduction (RRR) among cardiac surgery patients [10]. We made a more conservative assumption that mouthwash reduces pneumonia incidence by 0.3 RRR among abdominal surgery patients. In the absence of any other data, we extended the assumption to other parameters in mouthwash arm. Thus, we applied the 0.3 RRR assumption to critical care unit, mechanical ventilation (MV) and mortality probabilities. We conducted a range of sensitivity analysis around all the estimated parameters. The model probabilities are presented in Table 1.

**Table 1:** Model probabilities.

Parameter	Event	Total sample	Probability	Distribution	Source
**No-mouthwash-arm**					
**Patients with pneumonia**					
Pneumonia	39	931	0.04	Βeta	ASOS [1]
Critical care unit	21	39	0.54	Βeta	ASOS [1]
MV	-	-	0.25	Βeta	Expert Opinion
Died after MV	10	21	0.48	Βeta	ASOS [1]
Died (Critical care unit, No-MV)	17	42	0.40	Βeta	ASOS [1]
Died in general ward	3	75	0.04	Βeta	RECON [17]
**Patients with no pneumonia**					
Critical care unit	85	887	0.10	Βeta	ASOS [1]
MV	-	-	0.24	Fixed	Expert Opinion
Died after MV	-	-	0.04	Fixed	Expert Opinion
Died after no MV in critical care unit	29	85	0.34	Βeta	ASOS [1]
Died in general ward	25	802	0.03	Βeta	ASOS [1]
**Mouthwash arm**					
**Patients with pneumonia**					
Pneumonia	27	931	0.03	Βeta	ASOS [1] but reduced value in no-mouthwash arm by 0.3 RRR
Critical care unit	15	39	0.38	Βeta	ASOS [1], Reduced value in no-mouthwash arm by 0.3 RRR
MV	-	-	0.18	Fixed	Expert opinion, Reduced value in no-mouthwash arm by 0.3 RRR
Died after MV	7	21	0.33	Βeta	ASOS [1], Reduced value in no-mouthwash arm by 0.3 RRR
Died after no MV in critical care unit	12	42	0.28	Βeta	ASOS [1], Reduced value in no-mouthwash arm by 0.3 RRR
Died in general ward	3	75	0.04	Βeta	RECON [17], Assumed to be the same as in control arm
**Patients with no pneumonia**					
Critical care unit	85	887	0.10	Βeta	ASOS [1], Reduced value in no-mouthwash arm by 0.3 RRR
MV	-	-	0.24	Fixed	Expert opinion, assumed to be the same as in no-mouthwash-arm
Dead after MV	-	-	0.04	Fixed	Expert opinion, Assumed to be the same as in control arm
Dead (Critical care unit, No-MV)	29	85	0.34	Beta	ASOS [1], Assumed to be the same as in control arm
Dead in general ward	25	802	0.03	Βeta	ASOS [1] Assumed to be the same as in control arm

*MV = Mechanical ventilation.

#### Costs and resource use

Costs associated with postoperative pneumonia were sourced from studies done in USA through on a systematic review of the literature conducted as part of the current study (see S1 Paper). The estimated cost of treating a COVID-19 patient in South Africa was estimated by Davies et al (2019) [15]. However, costs for some parameters were not available in South Africa as such the mechanical ventilation cost was referenced from the UK National Health Service reference costs [16] and the cost of chlorhexidine was sourced from a UK private drug supplier [17].

To convert the UK and USA costs to South African values we adopted the market-basket estimation approach [18]. We collected the unit costs of a basket of healthcare goods and services: bed-day, critical care unit, outpatient care, drug and laboratory costs, laparotomy and radiological investigation available in UK and South Africa and then calculated the total cost of the basket [16, 19, 20]. The total cost of the basket in UK ($8,759), was divided by the total cost of the basket in South Africa ($4,519) to get the UK-South Africa Index (1.94). To convert the UK costs to South Africa costs we divided the cost of a good or service in UK by 1.94. The same method was used for a basket of bed-day, outpatient, drug and laboratory costs estimated by World Health Organisation (WHO) [19] to calculate the USA-South Africa index 10.8.

Costs in British Pounds were converted to International Dollars using purchasing power parity (PPP) conversion factors [21]. One study [20] did not specify the year of costs, hence we assumed that the last year of data collection (2015) was the year of the costs. All costs in the model were inflated to 2020 values [22] and assigned a gamma distribution. Model costs are presented in Table 2.

**Table 2:** Model input costs.

Resource use	Costs setting	Currency	Costs year	Unadjusted costs	Adjusted costs (2020 dollars)	Distribution	Source
Pneumonia	USA	US Dollar	2011	$23,030	$3,752	Gamma	Schmitges et al [23]
Bed day	South Africa	US Dollar	2008	$58	$71	Gamma	WHO [19]
ICU day	UK	British Pound	2015, assumed	£487	$1,210	Gamma	Dayananda et al [20]
Chlorhexidine (2 times)	UK	British Pound	2020	£8	$3	Gamma	Medisave [17]
Mechanical Ventilation	UK	British Pound	2018	£242	$89	Gamma	NHS [16]
Procedure cost	South Africa	British Pound	2015, assumed	£1,670	$1,423	Gamma	Dayananda et al [20]
COVID-19 cost per patient	South Africa	US Dollar	2019	£248	$254	Gamma	Davies et al [15]

#### Length of hospital stay

The LoS data were collected from the RECON dataset [14] (see Table 3). The LoS for the intervention and control arms were assumed to be identical for corresponding pathways.

**Table 3:** Length of Stay (LoS).

Parameter	Days (mean)	Source
Pneumonia, critical care unit	18.99	RECON [17]
Pneumonia, critical care unit, MV	21.12	RECON [17]
Pneumonia, critical care unit, No-MV	18.31	RECON [17]
Pneumonia, general ward	11.36	RECON [17]
No pneumonia, critical care unit	13.90	RECON [17]
No pneumonia, critical care unit, MV	15.32	RECON [17]
No pneumonia, critical care unit, No-MV	12.51	RECON [17]
No pneumonia, general ward	6.69	RECON [17]

*MV = mechanical ventilation.

#### Effectiveness measure

The effectiveness measure was the proportion of patients with pneumonia. Thus, the proportion of patients that had postoperative pneumonia in either the mouthwash or the no-mouthwash-surgery arm. In the analysis, an effectiveness value of “0” was assigned to pathways of patients who had postoperative pneumonia and a value of “1” to pathways of patients did not have postoperative pneumonia.

#### Analysis

The analysis took the South African healthcare provider perspective with costs presented in International Dollars ($) and South African Rands (R). We used a 30-day timeframe because evidence shows that readmission rates are negligible and rarely change within 30 days of surgery [24]. We assumed two scenarios:

- In Scenario 1, we assumed the absence of COVID-19. We assessed the costs and benefits of two times administration of preoperative 0.2% chlorhexidine mouthwash compared to no-mouthwash.
- In Scenario 2, the analysis was similar to scenario 1 but included the following assumptions on COVID-19: (i) COVID-19 will increase postoperative pneumonia probability by 1.8% as shown by the COVIDSurg dataset [8], (ii) in South Africa practices aimed at preventing hospital transmissions and managing COVID-19 infected patients will increase healthcare costs by $254 [15].

#### Deterministic sensitivity analysis

Deterministic sensitivity analysis (DSA) was conducted to assess the sensitivity of the base case results to changes in a given input parameter while holding constant the other parameters. In both scenarios 1 and 2, the conservative estimate on the RRR was reduced in various stages up to 0.01. At each RRR value we explored the cost of mouthwash gel that would change the base case results. The rest of the sensitivity analyses were conducted at a more conservative RRR value (0.01) as parameters would be more sensitive at a low RRR value. For the parameters estimated based on expert opinion and model assumptions, we varied the values further upwards and downwards up to 90% to explore the point where the base case results would change.

We additionally explored more scenarios assuming no patient needed mechanical ventilation and explored best case and worse case scenarios. For both scenarios 1 and 2, we simultaneously reduced probabilities of all mechanical ventilation pathways to zero to explore the possibility of abdominal surgery patient not requiring postoperative mechanical ventilation. Best and worst case scenario analyses were conducted by simultaneously changing a number of parameters using either the lower or upper bound values depending on what was likely to make things better or worse-off in terms of cost-effectiveness of mouthwash. The best case for mouthwash was when pneumonia, critical care unit, mechanical ventilation probabilities, general ward LoS, and critical care unit LoS were at the lower bounds and the bed day and pneumonia cost were at the upper bounds. Conversely, the lower bounds for bed day and pneumonia cost and the upper bounds for the former parameters were used as worst case scenario for mouthwash (see Table 4).

**Table 4:** Parameter values used in deterministic sensitivity analysis.

Variable	Base case value	Lower bound	Upper bound	Source
**Costs**				
Pneumonia cost	$3,752	$121	$5,804	Lower bound Eber et al, Upper bound Thompson et al [25, 26]
Bed day	$71	$57	$85	-20%, +20%
**Relative Risk Reduction**				
RRR	0.30	0.2, 0.10	0.05, 0.01	-10 percentage points, -10 percentage points, -5 percentage points, -4 percentage points
**No mouthwash**				
**Pneumonia patients**				
Pneumonia	0.04	0.03	0.05	-20%, +20%
Critical care unit	0.54	0.43	0.64	-20%, +20%
MV	0.25	0.10	0.40	-60%+60%
**No pneumonia patients**				
Critical care unit	0.10	0.08	0.11	-20%, +20%
MV	0.24	0.14	0.30	-40%+20%
Dead (critical care unit No-MV)	0.04	0.01	0.08	-80%, +90%
**No mouthwash, Mean LoS (days)**				
Pneumonia, critical care unit	18.99	15.19	22.79	-20%, +20%
Pneumonia, critical care unit, MV	21.12	16.90	25.34	-20%, +20%
Pneumonia, general ward	11.36	9.09	13.63	-20%, +20%
**Mouthwash**				
**Pneumonia patients**				
Pneumonia	0.04	0.03	0.05	-20%, +20%
Critical care unit	0.53	0.43	0.64	-20%+20%
MV	0.25	0.10	0.40	-60%, +60%
**No mouthwash patients**				
Critical care unit	0.10	0.08	0.11	-20%, +20%
MV	0.24	0.14	0.30	-40%+20%
Dead (critical care unit No-MV)	0.04	0.01	0.08	-80%, +90%
**Mouthwash, Mean LoS (days)**				
Pneumonia, critical care unit	18.99	15.19	22.79	-20%, +20%
Pneumonia, critical care unit, MV	21.12	16.90	25.34	-20%, +20%
Pneumonia, general ward	11.36	9.09	13.63	-20%, +20%
**Extreme cases**				
No patient on MV	-	-	-	Assumption
Best case scenario	-	-	-	Assumption
Worst case scenario	-	-	-	Assumption

*MV = mechanical ventilation.

#### Probabilistic sensitivity analysis

Probabilistic sensitivity analysis (PSA) was conducted to assess uncertainty of the base case estimates (in reference to 0.3 RRR). Ten thousand Monte Carlo simulations were run by simultaneously drawing random values of the parameters based on the assigned distribution. The results for each iteration were used to calculate the difference in costs and difference in proportion of pneumonia patients and presented on the cost-effectiveness plane [27]. We calculated the net benefits over a range of willingness to pay (WTP) thresholds per reduction in proportion of pneumonia patients and presented the results on cost-effectiveness acceptability curves (CEAC) [28, 29].

## Results

Results presented in Table 5 suggest that in the absence of COVID-19, the average hospital cost per patient was cheaper for mouthwash compared to no-mouthwash-surgery $3,675 (R 63,770) versus $3,958 (R 68,683) and mouthwash had lower proportion of pneumonia patients compared to no-mouthwash-surgery (0.029 versus 0.042) respectively. As such, mouthwash reduced hospital costs by $-283 (R -4,913) and reduced proportion of pneumonia patients by 0.013 implying that it dominated no-mouthwash-surgery (cheaper and more effective). In the presence of COVID-19, the results were similar to the no COVID-19 scenario, however, the presence of COVID-19 increased the difference in costs to $-303 (R -5,255) while the difference proportion of pneumonia patients remained at 0.013.

**Table 5:** Base case cost-effectiveness results.

Parameter	Scenario 1	Scenario 2
Mouthwash costs (USD)	3,675	3,698
No-mouthwash costs (USD)	3,958	4,001
Proportion of pneumonia patients (mouthwash)	0.029	0.031
Proportion of pneumonia patients (no-mouthwash)	0.042	0.045
Difference in costs (USD)	-283	-303
Difference in costs (R)	-4,913	-5,255
Difference (no-mouthwash-surgery minus mouthwash)in proportion of pneumonia patients	0.013	0.013

### Deterministic sensitivity analysis

#### Scenario 1

In the absence of COVID-19, only the analyses where either pneumonia, critical care unit, mechanical ventilation probabilities or critical care unit LoS in one arm was changed, holding constant the rest of the parameters, were any significant changes to base case results observed. A 20% increase in the probability of pneumonia patients admitted to critical care unit in the intervention arm made no-mouthwash-surgery dominant over mouthwash ($117 (R2,031) difference in costs and -0.0081 difference in proportion of pneumonia patients). When critical care unit LoS in the control arm was reduced from 18.99 to 15.19 days, mouthwash was more expensive but was slightly more effective which generated an ICER of $49,378 (R 856,802). The rest of the Scenario 1 DSA results are displayed in see Table 6.

**Table 6:** Scenario 1 deterministic sensitivity analysis results.

Parameter	Parameter value	Difference in costs ($)	Difference in costs (R)	Incremental benefits	Dominance break off point	ICER
**Base case**	N/A	-283	-4,913	0.0126	$289	Mouthwash dominates
**Relative Risk Reduction**						
RRR	0.20	-197	-3,423	0.0084	$203	Mouthwash dominates
	0.10	-101	-1,754	0.0042	$107	Mouthwash dominates
	0.05	-49	-851	0.0021	$55	Mouthwash dominates
	0.01	-6	-95	0.0004	$11	Mouthwash dominates
**Costs**						
Pneumonia	$121	-4	-69	0.0004		Mouthwash dominates
	$5,804	-6	-110	0.0004		Mouthwash dominates
Bed day	$57	-6	-96	0.0004		Mouthwash dominates
	$85	-5	-95	0.0004		Mouthwash dominates
**Probabilities**						
**No-Mouthwash**						
Pneumonia	0.03	167	2,895	-0.0115	N/A	No-mouthwash dominate
	0.05	-123	-2,135	0.0085	N/A	Mouthwash dominates
Pneumonia, critical care unit	0.43	95	1,642	0.0004	N/A	$225,863
	0.64	-99	-1,722	0.0004	N/A	Mouthwash dominates
Pneumonia, MV	0.10	4	65	0.0004	N/A	$8,962
	0.40	-15	-252	0.0004	N/A	Mouthwash dominates
No Pneumonia, critical care unit	0.08	273	4,743	0.0004	N/A	$652,472
	0.11	-284	-4,934	0.0004	N/A	Mouthwash dominates
No Pneumonia, MV	0.14	14	245	0.0004	N/A	$33,686
	0.30	-18	-314	0.0004	N/A	Mouthwash dominates
No Pneumonia, no MV, dead	0.01	-6	-95	0.0004	N/A	Mouthwash dominates
	0.08	-6	-95	0.0004	N/A	Mouthwash dominates
**Mouthwash**						
Pneumonia	0.03	-170	-2,956	0.0119	N/A	Mouthwash dominates
	0.05	117	2,031	-0.0081	N/A	No-mouthwash dominates
Pneumonia, critical care unit	0.43	-103	-1,786	0.0004	N/A	Mouthwash dominates
	0.64	92	1,595	0.0004	N/A	$219,374
Pneumonia, MV	0.10	-14	-251	0.0004	N/A	Mouthwash dominates
	0.40	3	60	0.0004	N/A	$8,312
No Pneumonia, critical care unit	0.08	-284	-4,936	0.0004	N/A	Mouthwash dominates
	0.11	273	4,745	0.0004	N/A	$652,763
No Pneumonia, MV	0.14	-25	-436	0.0004	N/A	Mouthwash dominates
	0.3	7	123	0.0004	N/A	$16,890
No Pneumonia, No MV, dead	0.01	-6	-95	0.0004	N/A	Mouthwash dominates
	0.08	-6	-95	0.0004	N/A	Mouthwash dominates
**Length of stay (mean days)**						
**No-Mouthwash**						
General ward	9.09	-2	-42	0.0004	7.34	Mouthwash dominates
	13.63	-9	-149	0.0004		Mouthwash dominates
Critical care unit	15.19	21	359	0.0004	18.19	$49,378
	22.79	-32	-550	0.0004		Mouthwash dominates
MV	16.9	-3	-58	0.0004	10.30	Mouthwash dominates
	25.34	-8	-133	0.0004		Mouthwash dominates
**Mouthwash**						
General ward	9.09	-9	-149	0.0004	15.38	Mouthwash dominates
	13.63	-2	-42	0.0004		Mouthwash dominate
Critical care unit	15.19	-31	-536	0.0004	19.81	Mouthwash dominates
	22.79	20	345	0.0004		$47,521
MV	16.9	-8	-132	0.0004	32.27	Mouthwash dominates
	25.34	-3	-59	0.0004		Mouthwash dominates
**Extreme case analysis**						
No patient on MV	N/A	-5	-88	0.0004	N/A	Mouthwash dominates
Best case scenario	N/A	-277	-4,801	0.0119	N/A	Mouthwash dominates
Worst case scenario	N/A	280	4,866	-0.0081	N/A	No-mouthwash dominates

*MV = mechanical ventilation.

Mouthwash dominated until general ward LoS was increased from 11.36 to 15.38 days or critical care unit LoS was increased from 18.99 to 19.81 days in the mouthwash arm or general ward LoS was decreased below 7.34 days or critical care unit LoS was decreased below 18.19 days in the no-mouthwash-surgery arm.

The extreme case analysis suggested that the cost of mouthwash gel had to increase at least to $11 (193) for mouthwash not to dominate. For example, at 0.03 and 0.01 RRR, mouthwash dominated no-mouthwash-surgery until the cost of mouthwash gel per patient rose above $289 (R 5,010) and $11 (R 193) respectively.

When no patient needed mechanical ventilation, mouthwash dominated as it reduced costs by $ -5 (R -88) and there was 0.0004 difference in proportion of pneumonia patients. In the best case scenario, mouthwash dominated as it reduced costs by $-277 (R -4,801) and the difference in proportion of pneumonia patients was 0.0119. In the worst case scenario, mouthwash was dominated with $280 (R 4,866) difference in costs and the difference in proportion of pneumonia patients was -0.0081.

#### Scenario 2

In the presence of COVID-19, DSA the results were similar to Scenario 1 results. Base case results changed only when either pneumonia, critical care unit, mechanical ventilation probabilities, general ward LoS or critical care unit LoS were varied in one arm (ceteris paribus). At 0.01 RRR, mouthwash dominated no-mouthwash-surgery as it reduced costs by $-6 (R –100) and the difference in proportion of pneumonia patients was 0.0004. The rest of the scenario 2 DSA results are displayed in Table 7.

**Table 7:** Scenario 2 deterministic sensitivity analysis results.

Parameter	Parameter value	Difference in costs (USD)	Difference in costs (R)	Incremental benefits	Dominance break off point	ICER
Base case	N/A	-303	-5,255	0.0134	$308	Mouthwash dominates
**Relative Risk Reduction**						
RRR	0.2	-211	-3,661	0.0090	$217	Mouthwash dominates
	0.1	-108	-1,874	0.0045	$114	Mouthwash dominates
	0.05	-52	-908	0.0022	$58	Mouthwash dominates
	0.01	-6	-100	0.0004	$11	Mouthwash dominates
**Costs**						
Pneumonia	$121	-4	-72	0.0004	N/A	Mouthwash dominates
	$5,804	-7	-116	0.0004	N/A	Mouthwash dominates
Bed day	$57	-6	-101	0.0004	N/A	Mouthwash dominates
	$85	-6	-100	0.0004	N/A	Mouthwash dominates
**Probabilities**						
**No-Mouthwash**						
Pneumonia	0.03	209	3,627	-0.0144	N/A	No-mouthwash dominates
	0.05	-81	-1,403	0.0056	N/A	Mouthwash dominates
Pneumonia, critical care unit	0.43	101	1,758	0.0004	N/A	$226,102
	0.64	-106	-1,840	0.0004	N/A	Mouthwash dominates
Pneumonia, mechanical ventilation	0.10	4	73	0.0004	N/A	$9,348
	0.40	-15	-268	0.0004	N/A	Mouthwash dominates
No Pneumonia, critical care unit	0.08	272	4,723	0.0004	N/A	$607,303
	0.11	-284	-4,924	0.0004	N/A	Mouthwash dominates
No Pneumonia, mechanical ventilation	0.14	14	239	0.0004	N/A	$30,731
	0.30	-18	-318	0.0004	N/A	Mouthwash dominates
No Pneumonia, no mechanical ventilation, dead	0.010	-6	-100	0.0004	N/A	Mouthwash dominates
	0.080	-6	-100	0.0004	N/A	Mouthwash dominates
**Mouthwash**						
Pneumonia	0.03	-212	-3,684	0.0148	N/A	Mouthwash dominates
	0.05	75	1,303	-0.0052	N/A	No-mouthwash dominates
Pneumonia, critical care unit	0.43	-107	-1,849	0.0004	N/A	Mouthwash dominates
	0.64	99	1,713	0.0004	N/A	$220,284
Pneumonia, MV	0.10	-15	-267	0.0004	N/A	Mouthwash dominates
	0.40	4	67	0.0004	N/A	$8,551
No Pneumonia, critical care unit	0.08	-284	-4,928	0.0004	N/A	Mouthwash dominates
	0.11	272	4,727	0.0004	N/A	$607,818
No Pneumonia, MV	0.14	-26	-448	0.0004	N/A	Mouthwash dominates
	0.30	6	109	0.0004	N/A	$13,963
No Pneumonia, no MV, dead	0.010	-6	-100	0.0004	N/A	Mouthwash dominates
	0.080	-6	-100	0.0004	N/A	Mouthwash dominates
**Length of stay (mean days)**						
**No Mouthwash**						
General ward	9.09	-2	-43	0.0004	7.41	Mouthwash dominates
	13.63	-9	-158	0.0004		Mouthwash dominate
Critical care unit	15.19	22	386	0.0004	18.21	$49,617
	22.79	-34	-587	0.0004		Mouthwash dominates
MV	16.90	-3	-60	0.0004	10.50	Mouthwash dominates
	25.34	-8	-140	0.0004		Mouthwash dominates
**Mouthwash**						
General ward	9.09	-9	-158	0.0004	15.30	Mouthwash dominates
	13.63	-2	-43	0.0004		Mouthwash dominates
Critical care unit	15.19	-33	-572	0.0004	19.80	Mouthwash dominates
	22.79	21	371	0.0004		$47,760
MV	16.90	-8	-139	0.0004	32.07	Mouthwash dominate
	25.34	-4	-62	0.0004		Mouthwash dominates
**Extreme case analysis**						
No patient on MV	N/A	-6	-101	0.0004	N/A	Mouthwash dominates
Best case scenario	N/A	-283	-4,917	0.0148	N/A	Mouthwash dominates
Worst case scenario	N/A	249	4,322	-0.0052	N/A	No-mouthwash dominate

*MV = mechanical ventilation.

#### Probabilistic Sensitivity Analysis (PSA)

**Fig 2:**
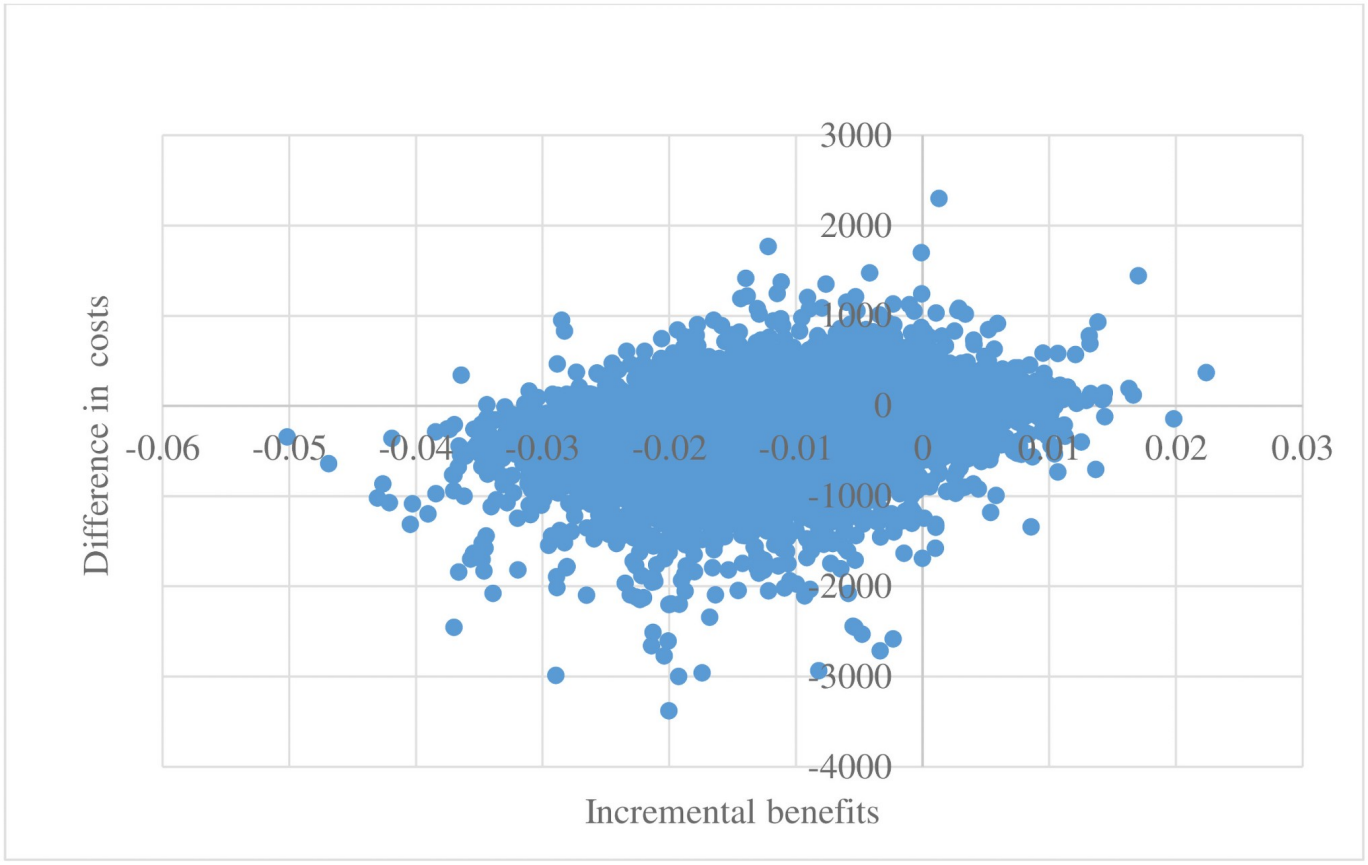
Cost-effectiveness scatter plot (in the absence of COVID-19).

The PSA results for scenario 1 are illustrated on the cost-effectiveness plane (see Fig 2). The average cost per patient for mouthwash was $5,067 (R 87,924) compared to no-mouthwash-surgery $5,302 (R 92,003). On average, mouthwash had lower proportion of pneumonia patients compared to no-mouthwash-surgery (0.028 versus 0.040). The mean difference in costs was $235 (R -4,079) and difference proportion of pneumonia patients was 0.012.

**Fig 3:**
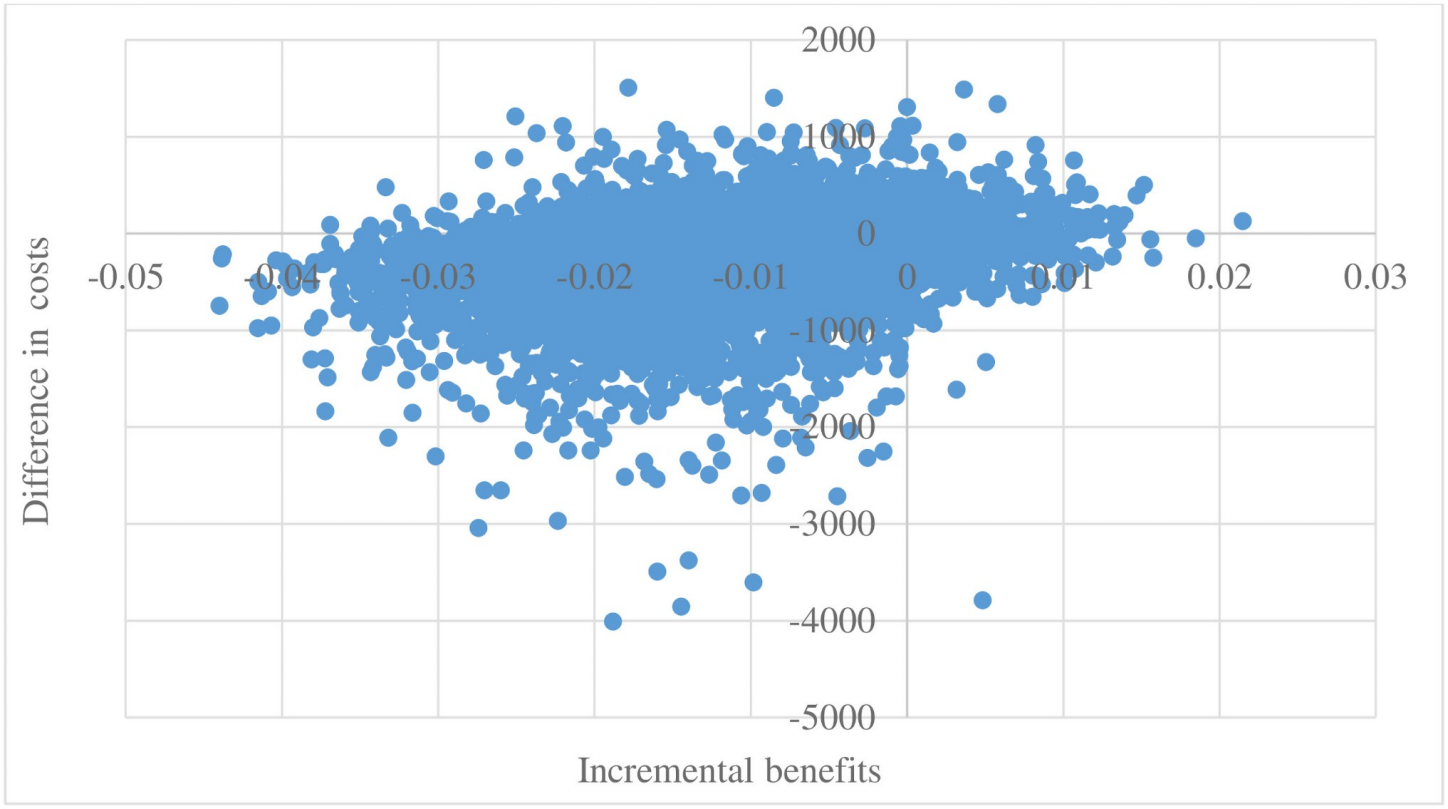
Cost-effectiveness scatter plot (COVID-19 period).

For scenario 2, the average difference in costs and proportion of pneumonia patients have been presented in Fig 3. The results suggest that mouthwash dominated no-mouthwash-surgery with $-276 (R -4,796) difference in costs and 0.012 difference in proportion of pneumonia patients.

**Fig 4:**
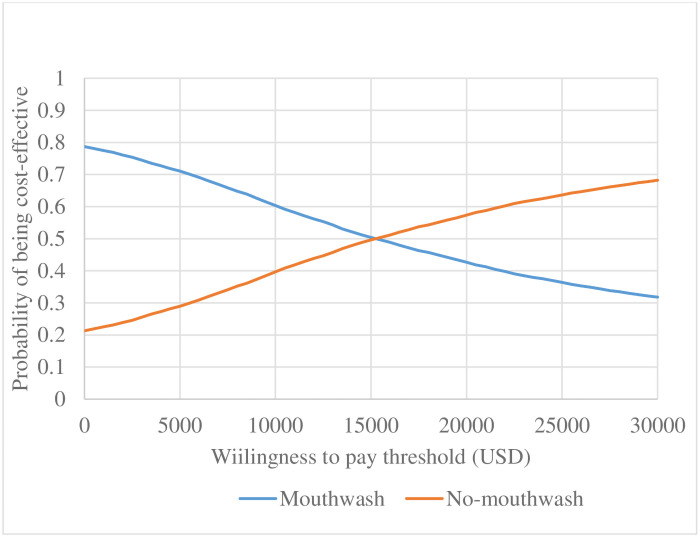
Cost-effectiveness acceptability curve (in the absence of COVID-19).

**Fig 5:**
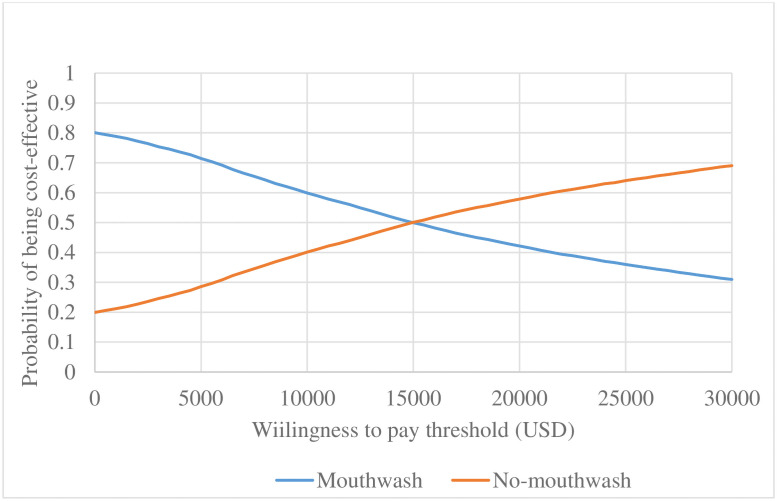
Cost-effectiveness acceptability curve (COVID-19 period).

Figs 4 and 5 present CEACs for scenarios 1 and 2 respectively with the WTP ranging from $0 (R 0) to $30,000 (R 520,560). In both the absence and presence of COVID-19, mouthwash was likely to be cost-effective until the WTP threshold was at $15,000 (R 260,280).

## Discussion

### Summary of findings

The results of the model-based analysis suggest that mouthwash is likely to dominate no-mouthwash-surgery in both the absence and presence of COVID-19. The average hospital costs for mouthwash were lower compared to no-mouthwash-surgery, at the same time, mouthwash was shown to have lower proportion of pneumonia cases than no-mouthwash-surgery. The results also show that there had to be a substantial reduction in general ward, critical care unit or mechanical ventilation LoS in the intervention arm or a substantial increase in at least one of the three parameters in the control arm for the intervention not to dominate (ceteris paribus). This implies that the small increase in costs associated with administering chlorhexidine mouthwash gel, $2.82 (R 18.85), is likely to be outweighed by the huge reduction in hospital costs arising from the reduction general ward, critical care unit and mechanical ventilation LoS.

The results also suggest that COVID-19 increases hospital costs as the difference in costs between mouthwash and no-mouthwash-arms became higher due to the presence of COVID-19. This suggests that as the number of postoperative pneumonia cases increase because of the COVID-19 pandemic it will be even more cost-saving for hospitals to use preoperative mouthwash since the intervention is likely to be associated with lower proportion of pneumonia cases than no-mouthwash-surgery thereby reducing hospital costs.

We made assumptions many of which deliberately undermined the possible effectiveness of mouthwash at reducing postoperative pneumonia. Despite the assumptions, our results have shown that the intervention is likely to be cost-effective. The extensive sensitivity analyses results suggested that even at low levels of effectiveness, mouthwash is likely to dominate no-mouthwash-surgery. The sensitivity analysis was conducted at a lower relative risk reduction rate suggesting that even the best case scenarios might have shown lower dominance than what could have been estimated if we had access to clinical data. This supports the urgent need to conduct clinical trials to access the effectiveness and cost-effectiveness of mouthwash surgery at reducing pneumonia among non-cardiac surgery patients to save resources especially LMICs.

### Strengths of the study

To our knowledge, this study is the first economic evaluation to explore the potential impact of preoperative mouthwash at reducing postoperative pneumonia at 30 days after abdominal surgery. Conducting research and randomised controlled trials in resource-limited settings is challenging because of the additional resources required to conduct such studies and collect robust data. The preliminary modelling approach carried out in this study has the benefit of highlighting the data which are likely to be most critical in the analysis and establish potential cost-effectiveness of preoperative mouthwash.

### Limitations

This study had several limitations specifically with respect to the dearth of available data.

First, three parameters in the control arm were estimated exclusively based on expert opinion. In the experimental arm, the critical care unit, mechanical ventilation and mortality probabilities of patients who developed pneumonia were estimated by reducing corresponding probabilities in the control arm assuming using 0.3 RRR. Further, in the experimental arm, critical care unit, mechanical ventilation and mortality probabilities of patients that did not develop pneumonia and the probability of dying in general ward for pneumonia patients were assumed to be same as probabilities of corresponding pathways in the control arm. On the positive side, the impact of the parameters on the model results were rigorously checked in the sensitivity analysis by reducing the RRR value to 0.01 and increasing or decreasing the probability of the parameter up to 90% and the results shown mouthwash to be a cost-effective intervention.

Second, using costs data from other countries was an inevitable limitation of the study. Finally, the objective of the analysis was to assess the impact of COVID-19 on increasing pneumonia incidence rates after surgery as such a transmission dynamic model may be considered more appropriate [30]. However, the intention was not to analyse the impact of COVID-19 on the hospital transmission of pneumonia but to capture just the increase in pneumonia incidence rates hence a non-transmission dynamic model was considered sufficient.

### Areas for future research

The model based analysis here was based on very limited primary data and heavily reliant on assumptions but ones which have deliberately attempted to undermine the benefit of mouthwash. Even in these scenarios the results suggest that mouthwash is likely to be cost saving. Thus, there is an urgent need to conduct appropriately focused clinical trials to assess the effectiveness and safety of the mouthwash intervention at reducing pneumonia among non-cardiac surgery patients in order to present robust evidence to support these results and to save crucial and scarce resources.

## Conclusions

The cost-effectiveness analysis was conducted to evaluate the potential cost-effectiveness of mouthwash at reducing pneumonia among abdominal surgery patients in South Africa. The results suggest that preoperative mouthwash surgery is likely to save money because it reduces LoS. Even a small improvement in effectiveness of mouthwash is likely to reflect good value for money. However, this analysis was based on numerous assumptions due to the paucity of evidence of the proposed intervention among non-cardiac surgery patients. Further research, in the form of a clinical trial is required to assess the effectiveness and safety of the intervention among non-cardiac surgery patients.

## Supporting information

S1 Appendix Questionnaire for estimating patient pathway probabilities. (DOCX)

S2 Appendix Full list of ASOS investigators and STARSurg collaborators. (DOCX)

S1 Paper Costs of post-operative pneumonia among abdominal surgery patients- a systematic review. (DOCX)

S1 Fig PRISMA flow diagram. (TIF)
