# Supplementary material for: Preliminary model assessing the cost-effectiveness of preoperative chlorhexidine mouthwash at reducing postoperative pneumonia among abdominal surgery patients in South Africa
Source: PLoS One. 2021 Aug 12;16(8):e0254698. doi: 10.1371/journal.pone.0254698 (PMC8360544; doi:10.1371/journal.pone.0254698)
Supplement: S1 Appendix — (DOCX) [file pone.0254698.s001.docx]

|  |  |  |  |  |  |  |  | Dead |  |
| --- | --- | --- | --- | --- | --- | --- | --- | --- | --- |
|  |  |  |  |  |  | Mechanical ventilation | |  |  |
|  |  |  |  |  |  | A3 |  |  |  |
|  |  |  |  | Critical Care Unit | |  |  | Discharged |  |
|  |  |  |  |  |  |  |  | Dead |  |
|  |  |  |  |  |  | No Mechanical ventilation | |  |  |
|  |  | Pneumonia |  |  |  |  |  | Discharged |  |
|  |  |  |  |  |  |  |  | Dead |  |
|  |  |  |  | General Ward |  |  |  |  |  |
|  |  |  |  |  |  |  |  | Discharged |  |
| Abdominal surgery | |  |  |  |  |  |  |  |  |
|  |  |  |  |  |  |  |  | Dead |  |
|  |  |  |  |  |  | Mechanical ventilation | | A2 |  |
|  |  |  |  |  |  | A1 |  |  |  |
|  |  |  |  | Critical Care Unit | |  |  | Discharged |  |
|  |  |  |  |  |  |  |  | Dead |  |
|  |  | No Pneumonia | |  |  | No Mechanical ventilation | |  |  |
|  |  |  |  |  |  |  |  | Discharged |  |
|  |  |  |  |  |  |  |  | Dead |  |
|  |  |  |  | General Ward | |  |  |  |  |
|  |  |  |  |  |  |  |  | Discharged |  |
|  |  |  |  |  |  |  |  |  |  |

The questions corresponds to the **no-mouthwash-arm** pathways A1, A2 and A3 highlighted in the model diagram above.

| **Question** | **Best Estimate** | **Lower limit estimate and Upper limit estimate** **(i.e. 10%-30%)** |
| --- | --- | --- |
| A1. Please estimate the percentage of patients that are **likely to require mechanical ventilation**  **To answer please**  consider abdominal surgery patients who:   - **Do not** acquire postoperative pneumonia, - **But** **are** admitted to Critical Care Unit. |  |  |
| A2. Please estimate the percentage of patients who are likely **to die before being discharged**  **To answer please** consider abdominal surgery patients who:   - **Do not** acquire postoperative pneumonia, - **But are** admitted to Critical Care Unit, - **And do** receive mechanical ventilation. |  |  |
| A3. Please estimate the percentage of patients that are **likely to require mechanical ventilation**  **To answer please**  consider abdominal surgery patients who:   - **Do** acquire postoperative pneumonia, - **Are** admitted to Critical Care Unit. |  |  |
