# Supplementary material for: Preliminary model assessing the cost-effectiveness of preoperative chlorhexidine mouthwash at reducing postoperative pneumonia among abdominal surgery patients in South Africa
Source: PLoS One. 2021 Aug 12;16(8):e0254698. doi: 10.1371/journal.pone.0254698 (PMC8360544; doi:10.1371/journal.pone.0254698)
Supplement: S2 Appendix — (DOCX) [file pone.0254698.s002.docx]

**GlobalSurg Collaborative Writing Committee**

Mwayi Kachapila, Adesoji O. Ademuyiwa, Bruce M. Biccard, Dhruva N. Ghosh, James Glasbey, Mark Monahan,Rachel Moore, Dion G. Morton, Raymond Oppong, Rupert Pearse, Tracy E. Roberts.

**ASOS Writing Committee**

Bruce M Biccard, Thandinkosi E Madiba, Hyla-Louise Kluyts, Dolly M Munlemvo, Farai Madzimbamuto, Apollo Basenero, Christina S Gordon, Coulibaly Youssouf, Sylvia Rakotoarison, Veekash Gobin, Ahmadou L Samateh, Chaibou M Sani, Akinyinka O Omigbodun, Simbo D Amanor-Boadu, Janat Tumukunde, Tonya M Esterhuizen, Yannick Le Manach, Patrice Forget, Abdulaziz M Elkhogia, Ryad M Mehyaoui, Eugene Zoumeno, Gabriel Ndayisaba, Henry Ndasi, Andrew Ndonga, Zipporah Ngumi, Ushmah Patel, Daniel Zemenfes Ashebir, Akwasi Antwi-Kusi, Bernard Mbwele, Hamza Doles Sama, Mahmoud Elfiky, Maher Fawzy, Rupert M Pearse, on behalf of the African Surgical Outcomes Study (ASOS) investigators.

**ASOS Steering Committee**

B M Biccard, T E Madiba, R M Pearse, on behalf of the African Surgical Outcomes Study (ASOS) investigators.

**ASOS investigators**

**Algeria:** National Leader: R Mehyaoui

Alger: R Mehyaoui, H Kanoun, L Chelbi, A Alghazali, A Metali (Établissement hospitalier spécialisé en chirurgie cardio-vasculaire Dr M.A Maouche ex CNMS); R Mokretar, F Ouanes (Centre Hospitalo Universitaire Issad Hassani, Beni-messous Alger); Z Imessaoudene, Khalfallah (Centre Hospitalo Universitaire Mustapha Pacha (Alger)); M Guenane, S Sadat (Centre Hospitalo Universitaire Salim Zemirli Alger); B Gadi, D Benali, N Chikh (Etablissement Hospitalo Universitaire d'Oran); M Laksari (Centre Hospitalo Universitaire Hassani Abdelkader, Sidi belabess); F Boufas (Etablissement Public Hospitalier Mostaganem).

**Benin**. National Leader: E Zoumenou

Joseph Akodjenou (Abomey-Calavi Hospital); Oswald Gbéhadé (Centre Hospitalier Départemental de l'Atacora); Serge Mewanou (Centre National Hospitalier Universitaire Hubert Koutoukou MAGA); Edith Djessouho (Centre Hospitalier Départemental de Mono/Couffo); Rene Ahossi (Centre Hospitalier Départemental de l’Ouémé-Plateau); Hippolyte Abadagan (Centre Hospitalier Départemental Zou); Ambroise Guegni (Centre Hospitalier Départemental-Donga); Guy Assoum (Hôpital de zone Sounon Séro de Nikki); Lidwine Zomahoun (Hôpital de la Mère et de l’Enfant Lagune de Cotonou); Kpatinvo Oscar (Hôpital de zone Aplahoue); Rosalie Souhe (Hôpital de zone de Boko); Hubert Dewanou (Hôpital de zone de Kandi); Rodrigue Nascimento (Hôpital de zone Menontin); Marius Guezo (Hôpital de zone Djougou (ordre de malte)); Sèhivè Valéry Adjignon (Hôpital de zone Malanville Karimama); Felix Anani (Hôpital de zone sanitaire Kouandé); Thomas Allaye (Hôpital de zone d'Adjohoun); Charles Tchegnonsi (Hôpital d'Instruction des Armées, Parakou), Lionelle Fanou (Hôpital d'Instruction des Armées de Cotonou); Rodrigue Hadecon (Hôpital de zone Banikoara), Anatole Atomabe (Hôpital de zone de Ouidah); Blaise Tchaou (University Departmental Hospital Borgou-Alibori, Parakou).

**Burundi. National Leader: G Ndayisaba**

E Horugavye (Bururi Hôpital); L Habimana (Clinique Prince Louis Rwagasore); T Nkesha (Hôpital Prince Regent Charles); C Manirimbere (Ngozi Hospital); R Ndikumana (Kamenge Military Hospital); JC Niyondiko (King Khaled Hospital of Bujumbura (CHUK)); P Minani (Kirundo Hospital); F Bigirimana (Regional Hospital of Gitega); JdD Uwiteyimbabazi (Ruyigi Hospital).

**Cameroon. National Leader: H Ndasi**

MA Degaulle (Banso Baptist Hospital); ND Konwuoh, G Ekwen; H Ndasi (Baptist Hospital Mutengene); M Kaggya, N Constance, N Samuel, S Akanyun, T Freeman (Mbingo Baptist Hospital); L Royas (Mboppi Baptist Hospital, Douala), M Tchoupa, WAG Simo, D Ngouane (Regional Hospital, Limbe); Shisong, BN Tanjong (Saint Elizabeth Catholic General Hospital).

**Congo.** National Leaders: AP Bouya, PDG Mawandza, Clinique Louise Michel, Pointe Noire:

MD Sedekounou, F Carlos, PD Gallou, A Prosper.

**Democratic Republic of Congo.** National Leader: Munlemvo DM

Athombo JS (Biamba Marie Mutombo Hospital); Sanduku DK. Mampangula Y (Centre de Sante Mater Dei); Munlemvo DM, Nziene VN (Centre Hospitalier et de Diagnostic Medical); Odia PK (Centre Hospitalier Grâce à Dieu); Monkole: Mbombo WD, Mbuyi WMS (Centre Hospitalier); Mukuna PM (Centre Medico-Chirurgical de Pigeon); Boloko PM, Mvwama NM, Ikuku JJN (Centre Mère et Enfant de Barumbu); Mwamba JD (Clinique Bondeko); Manzombi JK, Mvwala KR (Clinique Ngaliema); Ilunga JP.M, Mukenga MM, Mawisa T, Kilembe AM, Bikuelo CJ, Mwepu IM (Les cliniques universitaires de Kinshasa); Mwepu IM, Mwaluka CN, HN Sarah (El Rapha Clinic); Kukembila AM (Masi-Manimba Hospital); Diyoyo MP, Ngalala AM, Kabango R (Hôpital de L'amitie Sino-Congolaise, Njili); Kongolo M, Mbayabu M (Hôpital Dipumba); Namegabe ES, Bula Bula IM (Hôpital Général de Référence de N'djili); Likongo TB, Pengemale GN, Bingidimi SS, Imposo DH (Hôpital Général de Référence: Institut Médical Evangélique/kimpese); Matondo PM, Lelo SN (Hôpital Général de Référence De Kinkanda/Matadi); Kamanda RJ, N Kady (Hôpital Général de Référence De Makala); Ntueba B, Christian M (Hopital General De Reference Saint Luc De Kisantu); Badianga EK (Hôpital Presbytérien De Mbujimayi); Lubamba CLC (Hôpital Général de Référence: Jason Sendwe), Mubeya FK, Pembe JD, Katompwa PM (Hôpital Général de Référence: De Kinshasa); Yanga JJ, Lushima RF (Kapela Medical); Nantulu C, Shamamba R (Ngaliema Medical Center).

**Egypt.** National Leaders: MA Elfiky, M Fawzy

M Fawzy (Cairo University Hospitals); MA Elfiky (Cairo University Pediatric Hospital).

**Ethiopia.** National Leader: DZ Ashebir

A Berhe (ALERT Hospital); R Esayas (Ayder Referral Hospital); AB Zerihun, AT Robelie (Black Lion Hospital); MT Bahta (Jimma University Specialised Hospital); SK Kassa (Menelik II Hospital); SB Assefa (Myungsung Medical Center); GT Girmaye (Princes Zauditu Memorial Hospital).

**Gambia.** National Leader: AL Samateh

L Janneh, LN Sanyang (AFPRC General Hospital), MT Nyassi, SV Grey-Johnson (Bansang Hospital); R Villarreal, O Antùne (Bwiam Hospital); CS Jallow, CA Roberts, MF Aguilera Perez, MM Barow, M Bittaye, F Lanos, AL Samateh (Edward Francis Small Teaching Hospital); A Manneh, JTK Green-Harris (Serekunda General Hospital).

**Ghana.** National Leader: A. Antwi-Kusi

D Daliri (Bawku Presbyterian Hospital), AK Egote (Brong-Ahafo Regional Hospital, Sunyani); W Sam-Awortwi (Jnr) (Komfo Anokye Teaching Hospital); JN Clegg-Lamptey, YY Kutor, NA Adu-Aryee (Korle Bu Teaching Hospital); DP Domoyyeri (Koforidua Regional Hospital); E Atito-Narh (Ridge Regional Hospital); TWA Anabah, A Antwi-Kusi (Tamale Teaching Hospital).

**Kenya.** National Leaders: AKN Ndonga, ZWW Ngumi

C Bitta, HO Nyawanda, OEO Oduor, OS Okelo, SWO Ogendo (Jaramogi Oginga Odinga Teaching and Referral Hospital); NBM Ngari, NN Nyokabi (Karatina District Hospital); TM Chokwe, VM Gacii, B Githae, AP Gatheru, O Maranga, JM Muriithi, CM Mwangi, TM Mwiti, SM Nabulindo, C Ndung’u, K Ngugi, ZWW Ngumi, MN Njenga, S Njoroge, R Yahya, S Okiya, O Ogutu, DO Ojuka, PR Olang, J Mugambi, VM Mtongwe, B Kiprop, R Mbadi, PK Wambugu, S Hersi, S Mutahi, JS Aluvale, S Gitau, K Kariuki, E Ngatia, S Sitima, S Obadiah, V Mwenda, AM Iraya, EN Waweru, A Muturi, S Miima, M Ojujo, P Mossy, I Macharia, A Gakenia, F Mitema, M Manvinder, B Kipng’etich, M Njoroge, BO Semo, BM Ngari, D Ndegwa, JK Karanja, K Muchiri, O Makori, W Kibochi (Kenyatta National Hospital); W Mathangani, AKN Ndonga, A Swaleh, A Wafula (Mater Hospital); SN Maina, PW Njenga, N Okonu (Narok Level 4 Hospital).

**Libya.** National Leader: AM Elkhogia

M Maghrabi, A Jaber, MA Alkchr, H Agilla, M Bai, AAG Gomati (A Mansour Abu Salim Trauma Hospital); A Alsayed. Dr S Kafu, lM Milod, AM Mohamed, AA Aribi, EA Alzahra, MA Alzarouk, MM Ben Mansour, SS Saed, ES al Shams, MG el-Ghangouri, AS Shkirban, EA Alakkari, MG Gweder, RB Baghni, SA Alsabri, SH Hebli, M Elghandouri, SH Shanin, S Rokhsi, M EL Hensheri, M Eltaguri, IE Elyasir, HW Alwahedi, F Almoraid, N Anjar, A Alkilani, A Ghnain, FS Senusi (AL-Jala Obstetrics and Gynaecology Hospital, Tripoli); R Elsaity (Al Hawary Hospital, Benghazi); A Almajbery, A Abokris, L Montenegro, FAM Mohammed, H Alobeidi, MA Alomami, M Hammouda, G Elsaadi, NM Makhlouf, AZ Elzoway, TS Abuzalout, H Elmehdawi, A Elshikhy, MA Alhajj, A Alhendery Alhendery, A Tarhuni, A Feituri, ASB Benamour, F Alkobty Alkobty, HMZ Azzaidey, NM Mohammed, S Elfallah, ZS Elghazal, FFH (Alhoty Benghazi Medical Center); SOA Abugassa (El Khadra University Hospital), K Al-Zubaidy, M N Ilbarasi (Hawari General Hospital, Benghazi); R Alalem, E Swayeb, NZ Zubia, Dr Y Najm, P Mohamed, MO Teeka, T Mohamed, MSI Alayeb Alayeb, A Sohoub, A Abushnaf, A Mohamed, L Ramadan, A Shubba, O Huwidi, M Al-Serksi, M Eshtiwi, A Mohamed, E Hadia, A Mohamed, Al-J Ahmed, A Mohamed, I Mansour, JR Rian, G Yousef, S Mohamed, El-Z Ahmed, B Ahmed, S Mohamed, Z Jamal, Prof R Abusamra, SA Abujanah, Al-A Amer, O Yousef, W Abozaid, M A Elfagieh, A Ali, NZ Zoobei, A Juwid, A Wafa, Ortho A Alatresh, M Alashhab, AM Elzufri, AD Aldarrat, F Matoug, B Alsellabi, T Alkesa, A Addanfour, HB Badi, IE Elfaghi, E Salaheddin, L Hamadi (Misurata Cancer Center and Misurata Central Hospital); H Hashmi, A Abd-rouf , A Ali, M Ahmed, M Karar (National Cancer Institute, Sabratha); A Zidan, AA Alraheem, A Mohamed, A Attia, AA Yahya, F Otman, H Elmadany, G Gerbel, M Saleh, O Eurayet, R Almesmary, H Mostafa, S Abdurazig (Tobruk Medical Center); Dahim, K Jermi, MYK Koraz, AM Elghallal, AA Eshwehdi, A Elwerfelli, Dr A Elmgadmi, FT Elharati, D Elrafifi, M Algbali, F Abuhdema, L Elmadani, HA Algedar, I Shefren, A Abdulrahman, M Akkari, MS Sofia, NZ Najat, RM Elkhwildi, S Elgelany, A Alfetore, MSA Alshareef, A Abduljalil, A Hdud, HZ Zentuti, SB Khetrish, AZ Zeiton, AA Altagazi, SHA Almisslati, MG Gamal. YA Ali (Tripoli Medical Center); BA Albakosh, YG Gandy, I Jasim, IA Alghafoud, A Algddar, MA Alkassem, M Hokoma, SA Abusnina, M Gwila, AI Yahya*, M Mohammed, OA Alqawi, SH Hasan (Zliten Teaching Hospital).

**Madagascar.** National Leader: SR Rakotoarison

F Albert, AH Hery, RM Mamy (Centre Hospitalier de référence du Vakinankaratra); AGB Andriamampionona, RAF Rasolondraibe, MNR Mananjara, RR Rakotoarison, FAR Razanakoto, JA Hariniaina (Centre Hospitalier de Soaviandriana); A Rabemazava, DMA Randriambololona, DM Aurélia, M Judicaël, HE Ramilson, H Eloi, V Athanse, A Zo, L Andrianina, RM Judicael, AHN Rakotoarisoa, T François (Centre Hospitalier Universitaire Tanambao I d'Antsiranana); JDLC Rasolonjatovo, AHR Rakotoarijaona, MLR Ramananasoa, R Angelin, SR Rakotoarison, R Hanta (Centre Hospitalier Universitaire Analankininina Toamasina); RAR Raherison, FR Rija (Centre Hospitalier de Tambohobe); RAR Raherison, RF Fanjandrainy.(Hôpital Universitaire Joseph Ravoahangy Andrianavalona).

**Mali.** National Leader: Youssouf Coulibaly

Coulibaly Youssouf, Sanogo Zimogo Zié (Equipe d’investigateurs des CHU de Bamako CHU du Point G); Diallo Gangaly, Diango Djibo Mahamane (CHU Gabriel Touré); Touré Mamadou K, Simaga Abdoul K (CHU Luxembourg); Traoré Ibrahim, Traoré Abdoulaye (Equipe d’investigateur des Hôpitaux régionaux Hôpital de Mopti); Beye Seïdina Alioune (Hôpital de Ségou); Thiam Souleymane (Hôpital de Gao); Ongoïba Oumar (Hôpital de Tombouctou); Gaoussou Sogoba (Hôpital de Kayes); Maiga Amadou; (Kanté Moussa Hôpital de Sikasso).

**Mauritius.** National Leader: V Gobin

MD Maiyalagan, MA Cadersa, SSD Gaya (Apollo Bramwell Hospital); VMK Kissoon, J Gopall, FR Rajah, DM Mangoo, B Veerasamy, HMSN Heerah, ML Limbajee (Dr AG Jeetoo Hospital); SBM Alleesaib, TPR Ramchurn (Flacq Hospital); MTM Mandarry, S Joomye, V Gobin (Jawaharlall Nehru Hospital); B Thakoor, M Aungraheeta (Sir Seewoosagur Ramgoolam National Hospital); SS Sookun, SKB Boodhun (Subramania Bharathi Eye Hospital); MSR Rajcoomar, NB Babajee (Victoria Hospital).

**Namibia.** National Leaders: Apollo Basenero, CS Gordon

BS Sikombe (Engela State Hospital); KF Lumbala, K Ikandi, LT Kabongo (Gobabis State Hospital); M Garcia, P Izquirdo, AC Castro, IBB Ilunga, NAV Imbangu, F Nakandungile, JE Mmasi, O Tobiko, O Polishchuk, PNK Kashuupulwa, S Kalume, SE Sihope, O Arrey, SI Kakololo, NA Taiwo, PCK Kairuki, OGG Gazmuri, AM Imene, UA Umenushkin, A Kibandwa, JC Cisekedi, P Nakangombe , VEM Musiba, GGN Gama, HAM Mwanga, L Mgonja (Intermediate Hospital Oshakati); B Rink (Intermediate Hospital Katutura); BT Amisi, L Bangure, DS Sikazwe. OA Akinyemi, W Rois, I Rois (Katima Mulilo District Hospital); HK Sabwa (Keetmanshoop District Hospital); ML Barongo, SM Tshisekedi (Khorixas District Hospital); T Mbuyi (Luderitz State Hospital); TSK Lyimo (Mariental State Hospital); AA Munyika, AA Kornilov, PN Njuki, MA Ndaie, SSR Rwehumbiza (Onandjokwe Hospital); EK Makopa (Otjiwarongo District Hospital); W Njuguna, HP Mavesere (Outapi Hospital); AN Kabongo, MGH Mutasiigwa (Outjo District Hospital); YM Yangazov (Rundu Intermediate Referral Hospital); ATM Masiyambiri (St Martin's Hospital, Oshikuku); MJ Jannetjies, DF Dibwe, DV Manyere (St Mary’s Hospital, Rehobeth); O Gorelyk (Swakopmund State Hospital); K Kabangu, BNK Kaholongo, MA Amuthenu. Tsumeb District Hospital, S Stroyer. Walvis Bay District Hospital, B Rink, MA Labib, O Sosinska (Windhoek Central Hospital).

**Niger.** National Leader: Chaibou M Sani

A.Alfari (Maternity of Dosso); G Souley (Regional hospital of Agadez); AO Ousseini (Regional hospital of Maradi); M Amadou, AH.Harissou, MS Rabiou (National hospital of Zinder); MS Chaibou, Daddy H, MB Moussa Deye, Gagara M (National Hospital of Niamey); H Salaou (District hospital of Dakoro); M Moutari (Regional hospital of Diffa); I Bori (District hospital of Gaya); A Issoufou (District hospital of Mayahi); MS Dambaki (Regional hospital of Tahoua).

**Nigeria.** National Leader: Akinyinka O Omigbodun, Simbo D Amanor-Boadu

EO Ogboli-Nwasor, K Aghadi, I Mohammed, A Sarkin-Pawa, AA Yunus, Dr R Jimoh, AI Ibrahim, SA Edaigbini, Y Yakubu, T Sholadoye, AK Koledale (Ahmadu Bello University Teaching Hospital Zaria); LJC Anyanwu, M Atiku, HM Salisu-Kabara, AA Sheshe, SA Aji, AB Muhammad (Federal Medical Center, Owerri); NC Ekwunife, IAR Ike, A Anele, M Isiguzo, O Ihezie, Dr C Nnaji, A Onyegbule, EO Enendu (Aminu Kano Teaching Hospital, Kano); I Akhideno (Irrua Specialist Teaching Hospital, Irrua); IS Desalu, OB Bankole, RW Ojewola, AO Osinowo, BB Afolabi, ID Menkiti, BO Mofikoya (Lagos University Teaching Hospital, Idi Araba, Lagos); MR Mahmud, MO Osazuwa, AS Yusuf, YB Adamu (National Hospital, Abuja); OI Alatise, OA Arowolo, AO Adisa (Obafemi Awolowo University Teaching Hospitals Complex, Ile-Ife); IA Udo. KU Umeh, EB Etuk (University of Uyo Teaching Hospital, Uyo); OR Eyelade, O Ogunbode, OB Shittu, AO Omigbodun, OK Idowu, OO Ayandipo, TA Lawal, TO Ogundiran, TA Adigun, BB Osinaike, SD Amanor-Boadu (University College Hospital, Ibadan); OT Alagbe-Briggs, A Dodiyi-Manuel, RC Echem, J Otokwala, S Jamabo (University of Port Harcourt Teaching Hospital, Port Harcourt).

**Senegal.** National Leader: S Gueye

S Gueye (Centre Hospitalier Regional de Saint Louis).

**South Africa.** National Leader: H-L Kluyts

K Allopi, U Singh (Addington Hospital); JJA Bester, CR Saaiman (Beaufort West Hospital); KAP Bhat Z Nyatela-Akinrinmade (Cecilia Makiwane Hospital); A Alli, KE Bischof, H Hurri, OI Smith (Charlotte Maxeke Johannesburg Academic Hospital); AM Elghobashy, J Omoshoro-Jones (Chris Hani Baragwanath Academic Hospital); R Murray (Citrusdal Hospital); CH Basson (Dora Nginza hospital); DR Bhagwandass, MZ Koto (Dr George Mukhari Academic Hospital); DG Bishop, C Cairns, M Grobbelaar, TS Ngcobo NL Allorto, JD Bertie, A Dasrath, JB de Wet, GC Skinner, S Kransingh, W Kuhn, MP Hayes, ASM Madsen, JT Matubatuba, S Mayeza, LC Naidoo, S Jay Narain, T N Nkuebe, TN Shangase, RD Wise (Edendale Hospital); AJM Lumbamba, FK Mukinda (Eerste Rivier Hospital); M Coltman, JD Bruwer, L Nabela, M Nkunjana, S Mhatu, BP Thomas, EE Wynne (Frere Hospital), JG Davids, KK Grünewald, S Kruger, WAP Ras, PJ Venter (George Regional Hospital); SRC Cacala, Z Farina, V Govindasamy, C Kruse, C Lee, O Mahomva, LC Marais, S Rocher, RN Rodseth, R Sathiram, RP von Rahden, TD Naidoo, AS Singh (Greys Hospital); K Bhagwan, BM Biccard, ME Casey, E. Cloete, NL Fernandes (Groote Schuur Hospital); MAJ Jaworska (Helderberg Hospital); EM Semenya, SR Rayne (Helen Joseph Hospital); CS Alphonsus, T Biyase, MW Mbuyisa, AM Torborg, S Bechan, I Buccimazza, JT De Lange, LW Drummond, KD Dullab, BC Enicker, R Gigabhoy, R Goga, K Govender, TC Hardcastle, R Harrichandparsad, RE Hodgson, TK Kisten, BS Kusel, E Loots, A Maharaj, N Majola, VT Manzini, M Moopanar, IF Mukama, K Naicker, R Naidoo, TK Naidu, GK Ngcobo, M Phaff, H Ramnarain, SR Ramphal, AGL Rocher, N Sigcu, S Singh, HL Stoltenkamp, L Visser (Inkosi Albert Luthuli Hospital), ANJD Alberts, L Luthuli, FM Molokoane, OD Montwedi, TJ Rampai, H Schutte, DJ Van der Merwe, M Voigt (Kalafong); MAJ Jaworska (Karl Bremer Hospital), AJM Lumbamba (Khayelitsha Hospital); PGR Anderson, WM du Plessis, S Ellis, LM Ford, FJ Rousseau, N Rousseau, G Steyn, HC Steyn, M Weideman, B Church, EB Holford, AJ Szpytko (Kimberley Hospital Complex); NT Brouckaert, YBM Freeman (King DinuZulu Hospital); L Cronje, NZ Dube, A Ehouni, S Jithoo, L Naidoo, DL Skinner (King Edward VIII Hospital), A Mallier Peter, S Belhaj (Klerksdorp/Tshepong Hospital Complex), M Tun (Leratong Hospital); P Kapp (Livingstone hospital); YB Bwambale, H Holder, KJP Kasongo, V Makhanya, M Morapedi (Lower Umfolozi War Memorial Hospital); NA Mahomedy, V Pillay, SR Reddy, D Rungan, MB Greenwood, D Naidoo, R Randolph (Mahatma Gandhi Memorial Hospital); D Krick, J Roos (Mitchells Plain District Hospital), M Bester, D van Dyk (Mowbray Maternity Hospital); JD Bezuidenhout (Mseleni Hospital); BC Businge, BA Dokolwana, LP Jwambi, MM Liwani, N Mgoqo, B Mrara, Z Nibe, E Ninise, SM Pahlana, K Singata, MB Thomas, AO Usenbo, SM Malongwe (Nelson Mandela Academic Hospital); DG Giles, OS Porrill, AR Reed, H Steinhaus (New Somerset Hospital); MD Kabongo, MIS Kathrada, DA Maiwald, T Mniki (Northdale Hospital), MA Groenewald, J Visser (Oudtshoorn Hospital); GL Davies, VL Koller, QK Moodie, JM Spicer, JG Van Dyk (Paarl Provincial Hospital); BJS Diedericks, JL Esterhuizen, JJS van Niekerk (Pelonomi Hospital); MMZU Bhuiyan, R Mavhungu, TM Mhlari, M Akhter, AM Baloyi, KA Bamuza, MI Bamuza, L Bashiya, F Blignaut, A Bogoslovskiy, MM Chokoe, S Delinikaytis, BSG Diale, SN Dlamini, ME Gonzalez, TC Hlako, M Isaacs, MA Kolotsi, WJ Koperski, RM Lekoloane, B Lerutla, MT Lerutla, M Mabina, ME Maboya, AZ Machowski, LJ Madzivhe, SC Mafafo, RK Maila, DM Maimane, N Makgofa, MA Makwela, ME Malefo, DM Maluleke, MP Mamaleka, RS Masela, MM Mashamba, MV Mashile, OR Masia, JN Masipa, MW Matenchi, RC Mathe, JO Mogale, MP Mohlala, KN Mongwe, TG Morulana, SP Motloutsi, PE Mphephu, MP Muroonga, SZ Mzezewa, MC Neil, EV Neluheni, DS Nesengani, NT Nesengani, LE Netshimboni, I Notoane, S Omoding, O Orjiako, N Perez, RM Pheeha, KA Pilusa, MP Pochana, M Pupyshev, M Rambau, R Ramos, R Ramuntshi, H Roura, BB Ryabchiy, V Ryndine, MP Sebei, MP Segooa, MD Seshibe, GB Shah, V Sinoamadi, NLM Sithole, R Tshitangano, F Tshivhula (Polokwane Hospital); T Serdyn (Port Elizabeth Provincial Hospital); VL Moses, T Muzenda, N Naidoo, S van Wyk, L Rademan, A Garrido Lopez (Port Shepstone Hospital); DM Adeleke, E Blignaut, CM Human, K Kakudji, A Steyn, DI Uhuebor (Potchefstroom Hospital); JM Dippenaar (Pretoria Oral- and Dental Hospital); K Govender, ARW Mungherera, J Pansegrouw, RJ Ramjee (Prince Mshiyeni Memorial Hospital); EM Semenya (Rahima Moosa Mother and Child Hospital); A Ganesh, NF Rorke, N Abbas, V Dehal, P Govender, S Govender, C Houston, A Maharaj, AD Maharaj, A Nansook, MM Theunissen (RK Khan Hospital); C Lionnet, S Padayachee, T Ramsamy (St Aidans Mission Regional Hospital); K Kabongo, WP Kuhn, R Matos-Puig, A Thotharam, SK Tshisola, S Hariparsad, A Ibrahim Abdelatif, UV Jaganath, A Khamajeet, WP Makhaye, RD Naidoo, MB Patel (Stanger Hospital); L Dippenaar, HM Maakamedi, BM Mabaso, MR Maluleke, SR Motilall, LM Ntlhe, FP Paruk, BH Pienaar, S Spijkerman (Steve Biko Academic Hospital), M Brand, W Conradie, LJ du Preez, M Johnson, HM Ackermann, Y Baitchu, C Changfoot, MB Da Silva, DE du Plessis, D Hugo, AI Levin, M Mahoko, S Makhoba, JPB Maritz, S Naidoo, RM Rautenbach, D Roytowski, FH van der Merwe, AJ Vermeulen (Tygerberg Hospital), BJS Diedericks, GT Naude, D Tarloff (Universitas Hospital), BE Bilby, M Rademeyer (Vredenburg Hospital); B Dedekind, MI Hampton, JE van der Walt (Victoria Hospital), MS Nel (Witbank Hospital); R Duvenhage, HA van Zyl, CA Blake, EF Post, CAG Smits (Worcester hospital).

**Tanzania.** National Leaders: B Mbwele, P Forget

Said Gitta, B Mbwele (Bukombe District Hospital, Geita Tanzania); Henry Zepharine, John Igenge, Shaaban Nsalamba B Mbwele (Bukumbi Hospital); R Kalisa, P Waryoba, B Mbwele (Chato District Hospital); Ernest Nkwabi, John Lumona, Christopher Y Matola, Amina Kalisa, B Mbwele (Geita District Hospital); B Chrirangi, C Chalo, R Onyango, LVM Mesarieki, B Mbwele (Shirati Hospital).

**Togo.** National Leader: HD Sama

HD Sama (Sylvanus Olympio University Teaching Hospital).

**Uganda.** National Leader: JT Tumukunde

MTN Nabukenya (China-Uganda Friendship Hospital, Naguru), SCH Hodges (CoRSU Rehabilitation Hospital) FB Bulamba (CURE Children's Hospital Uganda), PKA Agaba (International Hospital Kampala), AFX Agaba, KJ Kuteesa, JK Kiwanuka, KL Kule (Kagando Hospital), GK Kateregga JK Kiwanuka (Mbarara Regional Referral Hospital), FB Bulamba, A Hewitt-Smith (Mbale Regional Referral Hospital), SA Senoga, HL Luweesi, MEK Knox (Mengo Hospital), AP Patience, KAM Kavuma, CN Namata, DK Kabatoro, IE Igaga, AET Ayebale, NP Nansubuga, OJP Ochieng, OM Othin, RN Nkwine, CS Sendagire, JT Tumukunde (Mulago Hospital); AK Kintu (Nakasero Hospital Limited).

**Zambia.** National Leader: Dr U. Patel

D.M. Linyama (Choma General Hospital); N. Nkuliyingoma, V.K. Kusweje, M.K. Kangili (Kabwe General Hospital); Mr Kalufwelu (Livingstone Central Hospital); A.M. Makupe, M Maimbo, Dr Kapesa, J.M. Musowoya (Ndola Central Hospital); N. Mbewe (Siavonga District Hospital); A. Mwale, C. Msadabwe, H. Nchimunya, N. Sipuka, P.D. Deka, S. Mwansa, Z. Rakhda, U. Patel (University Teaching Hospital).

**Zimbabwe.** National Leader: FD Madzimbamuto

E Chikumba, JT Chibanda (Baines Avenue Clinic); S Maweni (Belvedere Maternity Hospital);

CS Shambare (Bindura hospital); NK Chakafa, CJM Mawire (Chinhoyi Provincial Hospital); MC Chari, TM Mahureva, OM Moyo, PN Ndarukwa, MC Chiwanga (Chitugwiza Central Hospital); P Chimberengwa, K Dube, FK Mariwa (Gwanda Provincial Hospital); V Mushangwe (Gweru Provincial Hospital); HN Chifamba, T Saurombe (Harare Central Hospital); D Ngorora, CZ Zinyemba (Hwange Colliery Hospital); TW Munyaradzi (Kadoma General Hospital); PM Mapanda, M Hove(Kwekwe General Hospital); C Dhege, E Mashoko (Marondera Provincial Hospital); J Chirengwa (Masvingo Provincial Hospital), AM Macheka, A Mlotshwa (Mater Dei Hospital); C Ntoto, ND Chabayanzara, TNO Chaibva, HT Tshuma, (Mpilo Central Hospital), FM Chitungo (Mutare Provincial Hospital); GIM Muguti, M Mushaninga (Parirenyatwa Group of Hospitals); M Gova, S Zhou (PSMI Westend Hospital; G Mutizira (St Luke’s Hospital, Lupane); DM Mashava, L Katsukunya (The Avenues Clinic), EO Enwerem, T Gunguwo, K Kaseke (United Bulawayo Hospitals); S Ray, FFPH (RCP) Public Health (National planning group).

**STARSurg Collaborative** **Steering Committee**

**UK and Ireland** McLean KA (Study Lead), Khaw RA, Ahmed W, Akhbari M, Baker D, Borakati A, Kamarajah SK, Mills E, Murray V, Thavayogan R, Yasin I, Glasbey J (Guarantor).

**Australia** (TASMAN Collaborative): Raubenheimer K, Ridley W, Sarrami M, Zhang G, Egoroff N, Pockney P, Richards T.

**STARSurg Collaborative Advisory Group**

A Bhangu, B Creagh-Brown, M Edwards, EM Harrison, M Lee, D Nepogodiev, T Pinkney, P Pockney, R Pearse, T Richards, N Smart, R Vohra.

**STARSurg Collaborative** **Regional Leads**

| Sohrabi C (Barts and The London School of Medicine and Dentistry); Jamieson A (Brighton and Sussex Medical School); Nguyen M (Hull York Medical School); Rahman A (Imperial College London); English C (Ireland); Tincknell L (Kings College London); Kakodkar P (National University of Ireland Galway); Kwek I (Queen’s University Belfast); Punjabi N (St George’s, University of London); Burns J (University College Cork); Varghese S (University College Dublin and Trinity College Dublin); Erotocritou M (University College London); McGuckin S (University of Aberdeen); Vayalapra S (University of Birmingham); Dominguez E (University of Bristol); Moneim J (University of Cambridge); Bhatia S (University of Cardiff); Kouli O (University of Dundee); Salehi M (University of East Anglia); Tan HL (University of Edinburgh); Yoong A (University of Exeter); Zhu L (University of Glasgow); Seale B (University of Keele); Nowinka Z (University of Lancaster); Patel N (University of Leeds); Chrisp B (University of Leicester); Harris J (University of Limerick); Maleyko I (University of Liverpool); Muneeb F (University of Manchester); Gough M (University of Newcastle); James CE (University of Nottingham); Skan O (University of Oxford); Chowdhury A (University of Peninsula); Rebuffa N (University of Sheffield); Khan H (University of Southampton); Down B (University of Swansea); Fatimah Hussain Q (University of Warwick). |
| --- |

**STARSurg Collaborative** **authors (*denotes Hospital Lead)**

**Australia:** Adams M, Bailey A, Cullen G, Fu YXJ, McClement B, Taylor A* (Calvary Mater Hospital); Aitken S, Bachelet B, Brousse de Gersigny J, Chang C, Khehra B, Lahoud N, Lee Solano M, Louca M, Rozenbroek P, Rozenbroek P*, Rozitis E (Concord Repatriation Hospital); Agbinya N, Anderson E, Arwi G, Barry I, Batchelor C, Chong T, Choo LY, Clark L, Daniels M, Goh J, Handa A, Hanna J, Huynh L, Jeon A, Kanbour A, Lee A, Lee J, Lee T, Leigh J, Ly D, McGregor F, Moss J, Nejatian M, O’Loughlin E, Ramos I, Raubenheimer K, Richards T, Sanchez B, Shrivathsa A, Sincari A*, Sobhi S, Swart R, Trimboli J, Wignall P (Fiona Stanley Hospital); Bourke E, Chong A, Clayton S, Dawson A, Hardy E, Iqbal R, Le L, Mao S, Marinelli I, Metcalfe H, Panicker D, R HH, Ridgway S, Tan HH, Thong S, Van M, Woon S*, Woon-Shoo-Tong XS, Yu S (Gosford Hospital); Ali K, Chee J, Chiu C, Chow YW, Duller A, Nagappan P, Ng S, Pockney P, Selvanathan M, Sheridan C, Temple M* (John Hunter Hospital); Do JE, Dudi-Venkata NN, Humphries E*, Huynh L, Li L, Mansour LT, Massy-Westropp C (Royal Adelaide Hospital); Fang B, Farbood K, Hong H, Huang Y, Joan M, Koh C, Liu YHA, Mahajan T, Muller E*, Park R, Tanudisastro M, Wu JJG (Royal Prince Alfred Hospital); Chopra P*, Giang S, Radcliffe S, Thach P, Wallace D, Wilkes A (The Maitland Hospital); Chinta SH, Dawson A, Li J, Phan J, Rahman F, Segaran A, Shannon J, Woon-Shoo-Tong XS*, Zhang M (Wyong Hospital).

**Ireland:** Adams N, Bonte A, Choudhry A, Colterjohn N, Croyle JA, Donohue J, Feighery A, Keane A, McNamara D, Munir K*, Roche D, Sabnani R, Seligman D, Sharma S, Stickney Z, Suchy H, Tan R, Yordi S (Beaumont Hospital, Dublin ); Ahmed I, Aranha M, Burns J*, El Sabawy D, Garwood P, Harnett M, Holohan R, Howard R, Kayyal Y, Krakoski N, Lupo M, McGilberry W, Nepon H, Scoleri Y, Urbina C (Cork University Hospital); Ahmad Fuad MF, Ahmed O, Jaswantlal D, Kelly E, Khan MHT, Naidu D, Neo WX*, O’Neill R, Sugrue M (Letterkenny University Hospital); Abbas JD, Abdul-Fattah S, Azlan A, Barry K, Idris NS, Kaka N, Kakodkar P, Mc Dermott D, Mohammad Nasir MN, Mozo M, Rehal A, Shaikh Yousef M*, Wong RH (Mayo University Hospital); Curran E, Gardner M, Hogan A, Julka R*, Lasser G, Ní Chorráin N, Ting J (Portiuncula University Hospital); Browne R, George S, Janjua Z, Leung Shing V, Megally M, Murphy S, Ravenscroft L, Vedadi A, Vyas V* (Sligo Regional Hospital); Bryan A, Sheikh A, Ubhi J, Vannelli K, Vawda A* (South Tipperary General Hospital Clonmel); Adeusi L, Doherty C*, Fitzgerald C, Gallagher H, Gill P, Hamza H, Hogan M, Kelly S, Larry J, Lynch P, Mazeni NA, O’Connell R, O’Loghlin R, Singh K (St Vincent’s University Hospital); Abbas Syed R, Ali A, Alkandari B, Arnold A, Arora E, Azam R, Breathnach C*, Cheema J, Compton M, Curran S, Elliott JA, Hogan A, Jayasamraj O, Mohammed N, Noone A, Pal A, Pandey S, Quinn P, Sheridan R, Siew L, Tan EP, Tio SW, Toh VTR, Walsh M, Yap C, Yassa J, Young T (University Hospital Galway).

**United Kingdom:** Agarwal N, Almoosawy SA, Bowen K, Bruce D, Connachan R, Cook A, Daniell A, Elliott M, Fung HKF, Irving A, Laurie S, Lee YJ, Lim ZX, Maddineni S, McClenaghan RE, McGuckin S, Muthuganesan V, Ravichandran P, Roberts N, Shaji S, Solt S, Toshney E (Aberdeen Royal Infirmary); Arnold C, Baker O, Belais F, Bojanic C*, Byrne M, Chau CYC, De Soysa S, Eldridge M, Fairey M, Fearnhead N, Guéroult A, Ho JSY, Joshi K, Kadiyala N, Khalid S, Khan F, Kumar K, Lewis E, Magee J, Manetta-Jones D, Mann S, McKeown L, Mitrofan C, Mohamed T, Monnickendam A, Ng AYKC, Ortu A, Patel M, Pope T, Pressling S, Purohit K, Saji S, Shah Foridi J, Shah R, Siddiqui SS, Surman K, Utukuri M, Varghese A, Williams CYK, Yang JJ (Addenbrooke’s Hospital); Billson E, Cheah E, Holmes P, Hussain S, Murdock D, Nicholls A, Patel P*, Ramana G, Saleki M, Spence H, Thomas D, Yu C (Airedale General Hospital); Abousamra M, Brown C, Conti I, Donnelly A, Durand M*, French N, Goan R, O’Kane E, Rubinchik P (Altnagelvin Area Hospital); Gardiner H, Kempf B, Lai YL, Matthews H, Minford E, Rafferty C, Reid C*, Sheridan N (Antrim Area Hospital); Al Bahri T*, Bhoombla N, Rao BM, Titu L (Arrowe Park Hospital); Chatha S, Field C*, Gandhi T, Gulati R, Jha R, Jones Sam MT, Karim S, Patel R, Saunders M, Sharma K (Barnet General Hospital); Abid S*, Heath E, Kurup D, Patel A (Barnsley Hospital); Ali M, Cresswell B, Felstead D, Jennings K, Kaluarachchi T*, Lazzereschi L, Mayson H, Miah JE, Reinders B, Rosser A, Thomas C, Williams H (Basingstoke and North Hampshire Hospital); Al-Hamid Z, Alsadoun L, Chlubek M, Fernando P, Gaunt E, Gercek Y, Maniar R, Ma R, Matson M, Moore S, Morris A, Nagappan PG, Ratnayake M, Rockall L, Shallcross O, Sinha A, Tan KE, Virdee S, Wenlock R* (Bedford Hospital); Donnelly HA, Ghazal R, Hughes I, Lee J, Liu X, McFadden M, Misbert E, Mogey P, O’Hara A, Peace C, Rainey C, Raja P, Rubinchik P, Salem M*, Salmon J, Tan CH (Belfast City Hospital); Ahmed I, Alves D, Bahl S, Baker C*, Coulthurst J, Koysombat K, Linn T, Rai P, Sharma A, Shergill A (Blackpool Victoria Hospital); Ahmed M, Ahmed S, Belk LH, Choudhry H, Cummings D, Dixon Y, Dobinson C, Edwards J, Flint J, Franco Da Silva C, Gallie R, Gardener M*, Glover T, Greasley M, Hatab A, Howells R, Hussey T, Khan A, Mann A, Morrison H, Ng A, Osmond R, Padmakumar N, Pervaiz F, Prince R, Qureshi A, Sawhney R, Sigurdson B, Stephenson L, Vora K, Zacken A (Bradford Royal Infirmary); Cope P, Di Traglia R, Ferarrio I, Hackett N, Healicon R, Horseman L, Lam LI, Meerdink M*, Menham D, Murphy R, Nimmo I, Ramaesh A, Rees J, Soame R (Bristol Royal Infirmary); Dilaver N* (Bronglais Hospital); Adebambo D, Brown E, Burt J, Foster K*, Kaliyappan L, Knight P, Politis A, Richardson E, Townsend J (Calderdale Royal Hospital); Abdi M, Ball M, Easby S*, Gill N, Ho E, Iqbal H, Khan A, Matthews M, Nubi S, Nwokocha JO, Okafor I, Perry G, Reid C, Sinartio B, Vanukuru N, Walkley D, Welch T, Yates J, Yeshitila N (Castle Hill Hospital); Bryans K*, Campbell B, Gray C, Keys R, Macartney M (Causeway Hospital); Chamberlain G, Khatri A, Kucheria A, Lee STP, Reese G, Roy choudhury J, Tan WYR, Teh JJ*, Ting A (Charing Cross Hospital); Kazi S, Kontovounisios C, Vutipongsatorn K* (Chelsea and Westminster Hospital); Amarnath T, Balasubramanian N, Bassett E*, Gurung P, Lim J, Panjikkaran A, Sanalla A (Chesterfield Royal Hospital); Alkoot M, Bacigalupo V, Eardley N, Horton M, Hurry A, Isti C, Maskell P, Nursiah K, Punn G, Salih H (Countess of Chester Hospital); Epanomeritakis E, Foulkes A, Henderson R*, Johnston E, McCullough H, McLarnon M, Morrison E (Craigavon Area Hospital); Cheung A, Cho SH, Eriksson F, Hedges J, Khan F, Low Z, May C, Musto L, Nagi S, Nur S, Salau E*, Shabbir S, Thomas MC, Uthayanan L, Vig S, Zaheer M, Zeng G (Croydon Hospital); Ashcroft-Quinn S, Brown R, Hayes J, McConville R (Daisy Hill Hospital); French R*, Gilliam A, Sheetal S, Shehzad MU (Darlington Memorial Hospital); Bani W, Christie I, Franklyn J, Khan M, Russell J*, Smolarek S, Varadarassou R (Derriford Hospital); Ahmed SK, Narayanaswamy S, Sealy J, Shah M* (Doncaster Royal Infirmary); Dodhia V*, Manukyan A, O’Hare R, Orbell J (Dorset County Hospital, Dorchester); Chung I, Forenc K, Gupta A (Epsom Hospital); Agarwal A, Al Dabbagh A, Bennewith R, Bottomley J, Chu TSM*, Chu YYA, Doherty W, Evans B, Hainsworth P, Hosfield T, Li CH, McCullagh I, Mehta A, Thaker A, Thompson B, Virdi A, Walker H, Wilkins E (Freeman Hospital); Ali A, Dixon C, Hassan MR, Lotca N, Patel P, Tong KS (Furness General Hospital); Batchelor-Parry H, Chaudhari S, Harris T, Hooper J, Johnson C, Moss J, Mulvihill C, Nayler J, Olutobi O, Piramanayagam B, Stones K, Sussman M*, Weaver C (George Eliot Hospital); Alam F, Al Rawi M, Andrew F, Arrayeh A, Azizan N, Hassan A, Iqbal Z*, John I, Jones M, Kalake O, Keast M, Nicholas J, Patil A, Powell K, Roberts P, Sabri A, Segue AK, Shah A, Shaik Mohamed SA, Shehadeh A, Shenoy S, Tong A, Upcott M, Vijayasingam D (Glan Clwyd Hospital); Anarfi S, Dauncey J, Devindaran A*, Havalda P, Mwendwa E, Norman C, Richards J, Urquhart A (Glangwili General Hospital, Carmarthen); Allan J, Cahya E, Hunt H*, McWhirter C, Norton R, Roxburgh C, Tan JY (Glasgow Royal Infirmary); Ali Butt S, Hansdot S, Haq I, Mootien A*, Sanchez I, Vainas T (Glenfield Hospital); Deliyannis E*, Tan M, Vipond M (Gloucestershire Royal Hospital); Chittoor Satish NN, Dattani A, De Carvalho L*, Gaston-Grubb M, Karunanithy L, Lowe B, Pace C, Raju K, Roope J, Taylor C, Youssef H (Good Hope Hospital); Munro T, Thorn C, Wong KHF*, Yunus A (Great Western Hospital, Swindon); Chawla S, Datta A, Dinesh AA, Field D, Georgi T, Gwozdz A*, Hamstead E, Howard N, Isleyen N, Jackson N, Kingdon J, Patel A, Sagoo KS, Schizas A, Yin L (Guys and St.Thomas’ Hospital); Aung E, Aung YY, Franklin S, Han SM, Kim WC, Martin Segura A, Reese G, Rossi M, Ross T, Tirimanna R, Wang B*, Zakieh O (Hammersmith Hospital); Ali M, Ben-Arzi H, Flach A, Jackson E, Magers S, Olu abara C, Rogers E, Sharma K, Sugden K*, Tan H, Veliah S, Walton U (Harrogate District Hospital); Asif A, Bharwada Y*, Bowley D, Broekhuizen A, Cooper L, Evans N, Girdlestone H, Hussain S, Ling C, Mann H, Mehmood N, Mulvenna CL, Rainer N, Trout I (Heartlands Hospital, Birmingham); Gujjuri R, Jeyaraman D, Leong E, Leong E*, Leong E, Singh D, Smith E (Hereford County Hospital); Anderton J, Barabas M*, Goyal S, Howard D, Joshi A, Mitchell D, Weatherby T (Hinchingbrooke Hospital); Badminton R, Bird R, Burtle D, Choi NY, Devalia K, Farr E, Fischer F, Fish J, Gunn F*, Jacobs D, Johnston P, Kalakoutas A, Lau E, Loo YNAF, Louden H, Makariou N, Mohammadi K, Nayab Y, Ruhomaun S, Ryliskyte R, Saeed M, Shinde P, Sudul M, Theodoropoulou K, Valadao-Spoorenberg J, Vlachou F (Homerton Hospital); Arshad SR, Janmohamed AM, Murray V, Noor M, Oyerinde O, Saha A, Syed Y, Watkinson W (Huddersfield Royal Infirmary); Ahmadi H, Akintunde A, Alsaady A, Bradley J, Brothwood D, Burton M, Easby S*, Higgs M, Hoyle C, Katsura C, Lathan R, Louani A, Mandalia R, Nubi S, Nwokocha JO, Prihartadi AS, Qaddoura B, Sandland-Taylor L, Thadani S, Thompson A, Walkley D, Walshaw J (Hull Royal Infirmary); Teo S* (Inverclyde Royal Hospital); Ali S, Bawa JH, Fox S, Gargan K, Haider SA, Hanna N, Hatoum A, Khan Z, Krzak AM, Li T, Moneim J, Pitt J, Tan GJS, Ullah Z, Wilson E (Ipswich Hospital); Cleaver J, Colman J, Copeland L, Coulson A, Davis P, Faisal H, Hassan F, Hughes JT, Jabr Y, Mahmoud Ali F, Nahaboo Solim ZN, Sangheli A*, Shaya S, Thompson R (James Cook University Hospital); Cornwall H, De Andres Crespo M, Fay E, Findlay J*, Groves E, Jones O, Killen A, Millo J, Thomas S, Ward J, Wilkins M, Zaki F, Zilber E (John Radcliffe Hospital and Churchill Hospital); Bhavra K, Bilolikar A, Charalambous M, Elawad A*, Eleni A, Fawdon R, Gibbins A, Livingstone D, Mala D, Oke SE, Padmakumar D, Patsalides MA, Payne D, Ralphs C, Roney A, Sardar N, Stefanova K, Surti F, Timms R, Tosney G (Kettering General Hospital); Ali S, Bannister J, Clement NS*, Cullimore V, Kamal F, Lendor J, McKay J, Mcswiggan J, Minhas N, Seneviratne K, Simeen S, Taylor A, Valverde J, Watson N (King’s Mill Hospital); Bloom I, Dinh TH, Hirniak J, Joseph R, Kansagra M, Lai CKN*, Melamed N, Patel J, Randev J, Sedighi T, Shurovi B, Sodhi J, Vadgama N (Kingston Hospital); Abdulla S, Adabavazeh B, Champion A, Chennupati R, Chu K, Devi S, Haji A, Schulz J, Testa F* (King’s College Hospital); Davies P*, Gurung B, Howell S, Modi P, Pervaiz A, Zahid M (Leeds General Infirmary); Abdolrazaghi S, Abi Aoun R, Ali A, Anjum Z, Bawa G, Bhardwaj R, Brown S*, Enver M, Gill D, Gopikrishna D, Gurung D, Kanwal A, Kaushal P, Khanna A, Lovell E, McEvoy C, Mirza M, Nabeel S, Naseem S, Pandya K, Perkins R, Pulakal R, Ray M, Reay C, Reilly S, Round A, Seehra J, Shakeel NM, Singh B, Vijay Sukhnani M (Leicester General Hospital); Brown L, Desai B, Elzanati H, Godhaniya J, Kavanagh E, Kent J, Kishor A, Liu A, Naseem S*, Norwood M, Shaari N, Wood C, Wood M (Leicester Royal Infirmary); Agarwal A, Brown A, Chellapuri A, Ferriman A, Ghosh I, James CE, Kulkarni N, Noton T*, Pinto A, Rajesh S, Varghese B, Wenban C (Lincoln County Hospital); Aly R, Barciela C, Brookes T, Corrin E, Goldsworthy M, Mohamed Azhar MS, Moore J, Muneeb F, Nakhuda S, Ng D*, Pillay S, Port S (Manchester Royal Infirmary); Abdullah M, Akinyemi J, Islam S, Kale A*, Lewis A, Manjunath T, McCabe H, Misra S, Stubley T, Tam JP, Waraich N (Manor Hospital, Walsall); Chaora T, Ford C*, Khanna A, Osinkolu I, Pong G, Rai J, Risquet R (Milton Keynes University Hospital); Ainsworth J, Ayandokun P, Barham E, Barrett G, Barry J, Bisson E, Bridges I, Burke D, Cann J, Cloney M, Coates S, Cripps P, Davies C, Francis N, Green S, Handley G, Hathaway D*, Hurt L, Jenkins S, Johnston C, Khadka A, McGee U, Morris D, Murray R, Norbury C, Pierrepont Z, Richards C, Ross O, Ruddy A, Salmon C, Shield M, Soanes K, Spencer N, Taverner S, Williams C, Wills-Wood W, Woodward S (Morriston Hospital); Chow J*, Fan J, Guest O, Hunter I, Moon WY (Musgrove Park Hospital, Taunton); Arthur-Quarm S*, Edwards P, Hamlyn V, McEneaney L, N D G, Patil A, Pranoy S, Ting M (Nevill Hall); Abada S, Alawattegama LH*, Ashok A, Carey C, Gogna A, Haglund C, Hurley P, Leelo N, Liu B, Mannan F, Paramjothy K, Ramlogan K, Raymond-Hayling O, Shanmugarajah A, Solichan D, Wilkinson B (New Cross Hospital, Wolverhampton); Ahmad NA, Allan D, Amin A, Bakina C, Burns F, Cameron F, Campbell A, Cavanagh S, Chan SMZ, Chapman S, Chong V, Edelsten E, Ekpete O, El Sheikh M, Ghose R, Hassane A*, Henderson C, Hilton-Christie S, Husain M, Hussain H, Javid Z, Johnson-Ogbuneke J, Johnston A, Khalil M, Leung TCC, Makin I, Muralidharan V, Naeem M, Patil P, Ravichandran S, Saraeva D, Shafi S, Shankey-Smith W, Sharma N, Swan R, Waudby-West R, Wilkinson A, Wright K (Ninewells Hospital); Balasubramanian A, Bhatti S, Chalkley M, Chou WK, Dixon M, Evans L, Fisher K, Gandhi P, Ho S, Lau YB, Lowe S, Meechan C, Murali N, Musonda C, Njoku P, Ochieng L*, Pervez MU, Seebah K, Shaikh I, Sikder MA, Vanker R (Norfolk and Norwich University Hospital); Alom J*, Bajaj V, Coleman O, Finch G, Goss J, Jenkins C, Kontothanassis A, Liew MS, Ng K, Outram M, Shakeel MM, Tawn J (Northampton General Hospital); Green S, Zuhairy S* (North Durham University Hospital); Chapple K, Cinnamond A, Coleman S, George HA, Goulder L, Hare N, Hawksley J, Kret A, Luesley A, Mecia L*, Porter H, Puddy E, Richardson G, Sohail B, Srikaran V, Tadross D, Tobin J, Tokidis E, Young L (Northern General Hospital); Ashdown T, Bratsos S*, Koomson A, Kufuor A, Lim MQ, Shah S, Thorne EPC, Warusavitarne J, Xu S (Northwick Park / St. Mark’s Hospitals); Abigail S, Ahmed A, Ahmed J, Akmal A, Al-Khafaji M, Amini B, Arshad M, Bogie E, Brazkiewicz M, Carroll M, Chandegra A, Cirelli C, Deng A, Fairclough S, Fung YJ, Glover T, Gornell C, Green RL, Green SV, Gulamhussein AHM, Isaac AG, Jan R, Jegatheeswaran L, Knee M, Kotecha J*, Kotecha S, Maxwell-Armstrong C, McIntyre C, Mendis N, Naing TKP, Oberman J, Ong ZX, Ramalingam A, Saeed Adam A, Tan LL, Towell S, Yadav J (Nottingham City Hospital); Anandampillai R, Chung S, Hounat A*, Ibrahim B, Jeyakumar G, Khalil A, Khan UA, Kouli O, Nair G, Owusu-Ayim M, Wilson M (Perth Royal Infirmary); Kanani A, Kilkelly B, Ogunmwonyi I, Ong L, Samra B, Schomerus L, Shah R, Shea J, Turner O, Yang Y* (Peterborough City Hospital); Amin M, Blott N, Clark A, Feather A, Forrest M, Hague S, Hamilton K, Higginbotham G, Hope E, Karimian S, Loveday K, Malik H, McKenna O, Noor A, Onsiong C, Patel B, Radcliffe N, Shah P, Tye L, Verma K, Walford R*, Yusufi U, Zachariah M (Pinderfields Hospital); Casey A, Doré C, Fludder V, Fortescue L, Kalapu SS, Karel E, Khera G, Smith C (Princess Royal Hospital, Haywards Heath); Appleton B, Ashaye A, Boggon E, Evans A, Faris Mahmood H, Hinchcliffe Z, Marei O, Silva I, Spooner C, Thomas G, Timlin M, Wellington J*, Yao SL (Princess of Wales, Bridgend); Abdelrazek M, Abdelrazik Y, Bee F, Joseph A, Mounce A, Parry G, Vignarajah N* (Prince Charles Hospital, Merthyr); Biddles D, Creissen A, Kolhe S, K T, Lea A*, Ledda V, O’Loughlin P, Scanlon J, Shetty N, Weller C (Queen Elizabeth Hospital, Gateshead); Abdalla M*, Adeoye A, Bhatti M, Chadda KR, Chu J, Elhakim H, Foster-Davies H, Rabie M, Tailor B, Webb S (Queen Elizabeth Hospital King’s Lynn); Abdelrahim ASA, Choo SY*, Jiwa A, Khan M, Mangam S, Murray S, Shandramohan A, Tincknell L (Queen Elizabeth The Queen Mother Hospital, Margate); Aghanenu O, Budd W, Gornell C, Hayre J, Khanom S, Liew ZY, Maxwell-Armstrong C, McKinney R, Moody N*, Muhammad-Kamal H, Odogwu J, Patel D, Roy C, Sattar Z, Shahrokhi N, Sinha I, Thomson E, Wonga L (Queens Medical Centre, Nottingham); Bain J, Khan J, Ricardo D* (Queen Alexandra Hospital, Portsmouth); Bevis R, Cherry C, Darkwa S, Drew W, Griffiths E, Konda N, Madani D, Mak JKC, Meda B, Odunukwe U, Patel J, Preest G, Raheel F, Rajaseharan A, Ramgopal A, Risbrooke C, Selvaratnam K, Sethunath G, Sharma S*, Tabassum R, Taylor J, Thakker A, Wijesingha N, Wybrew R, Yasin T (Queen Elizabeth Hospital, Birmingham); Ahmed Osman A, Alfadhel S, Carberry E, Chen JY*, Drake I, Glen P, Jayasuriya N, Kawar L, Myatt R, Sinan LOH, Siu SSY, Tjen V (Queen Elizabeth University Hospital, Glasgow); Adeboyejo O, Bacon H, Barnes R, Birnie C, D’Cunha Kamath A, Hughes E, Middleton S, Owen R*, Schofield E, Short C, Smith R, Wang H, Willett M, Zimmerman M (Royal Berkshire Hospital); Balfour J, Chadwick T, Coombe-Jones M, Do Le HP, Faulkner G, Hobson K*, Shehata Z (Royal Bolton Hospital); Beattie M*, Chmielewski G, Chong C, Donnelly B, Drusch B, Ellis J, Farrelly C, Feyi-Waboso J, Hibell I, Hoade L, Ho C, Jones H, Kodiatt B, Lidder P, Ni Cheallaigh L, Norman R, Patabendi I, Penfold H, Playfair M, Pomeroy S, Ralph C, Rottenburg H, Sebastian J, Sheehan M, Stanley V, Welchman J (Royal Cornwall Hospital); Ajdarpasic D, Ali S, Antypas A, Azouaghe O, Basi S, Bettoli G, Bhattarai S, Bommireddy L*, Bourne K, Budding J, Cookey-Bresi R, Cummins T, Davies G, Fabelurin C, Gwilliam R, Hanley J, Hird A, Kruczynska A, Langhorne B, Lund J, Lutchman I, McGuinness R, Neary M, Pampapathi S, Pang E, Podbicanin S, Rai N, Redhouse White G, Sujith J, Thomas P, Walker I, Winterton R (Royal Derby Hospital); Anderson P, Barrington M, Bhadra K, Clark G, Fowler G, Gibson C, Hudson S, Kaminskaite V, Lawday S*, Lee M, Longshaw A, MacKrill E, McLachlan F, Murdeshwar A, Nieuwoudt R, Parker P, Randall R, Rawlins E, Reeves SA, Rye D, Sirkis T, Smart N, Sykes B, Ventress N, Wosinska N, Yoong A (Royal Devon and Exeter Hospital); Akram B, Burton L, Coombs A*, Long R, Magowan D, Ong C, Sethi M, Williams G (Royal Gwent Hospital, Newport); Cambridge W, Chan C, Chan LH, Fernando D, Gaba F*, Khor Z, Les JW, Mak R, Moin S, Ng Kee Kwong KC, Paterson-Brown S, Tew YY (Royal Infirmary of Edinburgh); Bardon A, Burrell K, Coldwell C, Costa I, Dexter E, Hardy A, Khojani M, Mazurek J, Nowinka Z, Patel D, Raymond T, Reddy V, Reynolds J, Soma A (Royal Lancaster Infirmary); Agiotakis S*, Alsusa H, Desai N, Peristerakis I (Royal Preston Hospital); Adcock A, Ali S, Ayub H, Bennett T, Bibi F, Brenac S, Chapman T, Clarke G, Clark F, Galvin C, Gwyn-Jones A, Henry-Blake C, Kerner S, Kiandee M*, Lovett A, Pilecka A, Ravindran R, Seale B, Siddique H, Sikand T, Treadwell K (Royal Stoke University Hospital); Ahmed S, Akmal K, Apata A, Barton O, Broad G, Darling H, Dhuga Y, Emms L, Fludder V, Habib S, Jain R, Jamieson A, Jeater J, Kan CYP, Kathiravelupillai A, Khatkar H, Khera G, Kirmani S, Kulasabanathan K, Lacey H, Lal K, Manafa C, Mansoor M*, McDonald S, Mittal A, Mustoe S, Nottrodt L, Oliver P, Papapetrou I, Pattinson F, Raja M, Reyhani H, Shahmiri A, Small O, Soni U (Royal Sussex County Hospital); Aguirrezabala Armbruster B, Bunni J, Hakim MA*, Hawkins-Hooker L, Howell KA, Hullait R, Jaskowska A, Ottewell L, Thomas-Jones I, Vasudev A (Royal United Hospital, Bath); Ashcroft-Quinn S, Clements B, Donnelly HA, Fenton J, Gill M, Haider S, Lim AJM, Maguire H, McMullan J, Nicoletti J, Samuel S, Unais MA, White N, Yao PC, Yow L (Royal Victoria Hospital, Belfast); Boyle C*, Brady R, Browne R, Cheekoty P, Cheong J, Chew SJHL, Chow R, Ganewatta Kankanamge D, Mamer L, Mohammed B, Ng Chieng Hin J, Renji Chungath R, Royston A, Sharrad E, Sinclair R, Tingle S, Treherne K, Wyatt F (Royal Victoria Infirmary, Newcastle); Edwards J, Maniarasu VS*, Moug S (Royal Alexandra Hospital, Paisley); Appanna T, Bucknall T, Hussain F, Khan A*, Owen A, Parry M, Parry R, Sagua N, Spofforth K, Yuen ECT (Royal Glamorgan Hospital); Bosley N, Hardie W, Moore T, Regas C* (Royal Hampshire Hospital, Winchester); Abdel-Khaleq S, Ali N, Bashiti H, Buxton-Hopley R, Constantinides M, D’Afflitto M, Deshpande A, Duque Golding J, Frisira E, Germani Batacchi M, Gomaa A, Hay D, Hussain S, Hutchison R, Iakovou A, Iakovou D, Ismail E, Jefferson S, Jones L, Khouli Y, Knowles C, Liu X*, Mason J, McCaughan R, Moffatt J, Morawala A, Nadir H, Neyroud F, Nikookam Y, Parmar A, Pinto L, Ramamoorthy R, Richards E, Shah R, Thomson S, Trainer C, Valetopoulou A, Vassiliou A, Wantman A, Wilde S (Royal London Hospital); Dickinson M, Rockall T, Senn D, Wcislo K*, Zalmay P (Royal Surrey Hospital, Guildford); Adelekan K, Ahmed A, Allen K, Bajaj M, Gatumbu P, Hang S, Hashmi Y, Kaur T, Kawesha A, Kisiel A*, Woodmass M (Russell’s Hall Hospital); Adelowo T, Ahari D, Alhwaishel K, Atherton R, Clayton B, Cockroft A, Curtis Lopez C, Hilton M, Ismail N, Kouadria M, Lee L, MacConnachie A, Monks F, Mungroo S, Nikoletopoulou C, Pearce L, Sara X*, Shahid A, Suresh G, Wilcha R (Salford Royal Infirmary); Atiyah A, Brown L, Davies E, Dermanis A, Gibbons H, Hyde A, Lawson A, Lee C, Leung-Tack M, Li Saw Hee J, Mostafa O, Nair D, Pattani N, Plumbley-Jones J, Pufal K, Ramesh P, Rogers E, Sanghera J, Saram S, Scadding S, See S*, Stringer H, Torrance A, Vardon H, Vayalapra S, Wyn-Griffiths F (Sandwell General Hospital); Brew A, Kaur G, Soni D*, Tickle A (Scunthorpe General Hospital); Akbar Z, Appleyard T, Figg K, Jayawardena P*, Johnson A, Kamran Siddiqui Z, Lacy-Colson J, Oatham R, Rowlands B, Sludden E, Turnbull C (Shrewbury and Telford Hospitals); Allin D, Ansar Z, Azeez Z, Barry J, Dale VH, Garg J, Horner A, Jones S, Knight S, McGregor C, McKenna J, McLelland T, Packham-Smith A, Rowsell K, Spector-Hill I* (Singleton Hospital); Adeniken E, Baker J, Bartlett M, Chikomba L, Chong C, Connell B, Deekonda P, Dhar M, Elmansouri A, Gamage K, Goodhew R, Hanna P, Jones L, Knight J, Luca A, Maasoumi N*, Mahamoud F, Manji S, Marwaha PK, Mason F, Oluboyede A, Pigott L, Razaq AM, Richardson M, Saddaoui I, Wijeyendram P, Yau S (Southampton General Hospital); Atkins W*, Liang K, Miles N, Praveen B, Rajesh S (Southend University Hospital); Ashai S, Braganza J, Common J, Cundy A, Davies R, Guthrie J, Handa I, Iqbal M, Ismail R, Jones C*, Jones I, Lee KS, Levene A, Okocha M, Olivier J, Smith A, Subramaniam E, Tandle S, Wang A, Watson A, Wilson C (Southmead Hospital, Bristol); Chan XHF, Khoo E, Lim J, Montgomery C, Norris M, Pugalenthi PP (South West Acute Hospital); Common T, Cook E, Mistry H*, Shinmar HS (St Mary’s Hospital IOW); Agarwal G, Bandyopadhyay S, Brazier B, Carroll L, Goede A, Harbourne A, Lakhani A, Lami M, Larwood J, Martin J, Merchant J*, Pattenden S, Pradhan A, Raafat N, Rothwell E, Shammoon Y, Sudarshan R, Vickers E, Wingfield L (Stoke Mandeville Hospital); Ashworth I, Azizi S, Bhate R, Chowdhury T, Christou A, Davies L, Dwaraknath M, Farah Y, Garner J, Gill N, Gureviciute E, Hart E, Jain A, Javid S, Kankam HK, Kaur Toor P, Kaz R*, Kermali M, Khan I, Khan M, Mattson A, McManus A, Murphy M, Nair K, Ngemoh D, Norton E, Olabiran A, Parry L, Payne T, Pillai K, Price S, Punjabi K, Raghunathan A, Ramwell A, Raza M, Ritehnia J, Simpson G, Smith W, Sodeinde S, Studd L, Subramaniam M, Thomas J, Towey S, Tsang E, Tuteja D, Van M, Vasani J, Vio M (St. Georges Hospital); Badran A*, Gupta A (St. Helier Hospital); Adams J, Anthony Wilkinson J, Asvandi S, Austin T, Bald A, Bix E, Carrick M, Chander B, Chowdhury S, Cooper Drake B, Crosbie S, D. Portela S, Francis D, Gallagher C, Gillespie R, Gravett H, Gupta P, Ilyas C*, James G, Johny J, Jones A, Kinder F, MacLeod C, Macrow C, Maqsood-Shah A, Mather J, McCann L, McMahon R, Mitham E, Mohamed M, Munton E, Nightingale K, O’Neill K, Onyemuchara I, Patel R, Senior R, Shanahan A, Sherlock J, Spyridoulias A, Stavrou C, Stokes D, Tamang R, Taylor E, Trafford C, Uden C, Waddington C, Yassin D, Zaman M (St James University Hospital, Leeds); Bangi S, Bratsos S, Cheng T, Chew D, Hussain N, Imani-Masouleh S, Mahasivam G, McKnight G, Ng HL, Ota HC, Pasha T, Ravindran W, Reese G, Shah K, Vishnu K S*, Xu S, Zaman S (St. Mary’s Hospital, London); Carr W, Cope S, Eagles EJ, Howarth-Maddison M, Jones S, Li CY, Reed J, Ridge A, Stubbs T*, Teasdaled D, Umar R, Worthington J (Sunderland Royal Hospital); Dhebri A, Kalenderov R* (Tameside Hospital); Alattas A, Arain Z, Bhudia R*, Chia D, Daniel S, Dar T, Garland H, Girish M, Hampson A, Kyriacou H, Lehovsky K, Mullins W, Omorphos N, Vasdev N, Venkatesh A, Waldock W (The Lister Hospital, Stevenage); Bhandari A, Brown G, Choa G, Eichenauer CE, Ezennia K, Kidwai Z, Lloyd-Thomas A, Macaskill Stewart A*, Massardi C, Saunders M, Shah S, Sinclair E, Skajaa N, Smith M, Tan I (The Royal Free Hospital); Afsheen N, Anuar A, Azam Z, Bhatia P, Davies-kelly N, Dickinson S, Elkawafi M, Ganapathy M, Gupta S, Khoury EG*, Licudi D, Mehta V, Neequaye S, Nita G, Tay VL, Zhao S (The Royal Liverpool University Hospital); Botsa E, Cuthbert H, Elliott J, Furlepa M, Lehmann J, Mangtani A, Narayan A, Nazarian S*, Parmar C, Shah D, Shaw C, Tan M, Zhao Z (The Whittington Hospital); Beck C*, Caldwell S, Clements JM, French B, Kenny R, Kirk S, Lindsay J, McClung A, McLaughlin N, Watson S, Whiteside E (Ulster Hospital); Ahmed A, Alyacoubi S*, Arumugam V, Beg R, Dawas K, Garg S, Lloyd ER, Mahfouz Y, Manobharath N, Moonesinghe R, Morka N, Patel K, Prashar J, Tan M, Yip S (University College Hospital); Adeeko ES, Ajekigbe F, Bhat A, Evans C, Farrugia A, Gurung C, Long T, Malik B*, Manirajan S, Newport D, Qureshi A, Rayer J, Ridha A, Ross E, Saran T, Sinker A, Smith M, Waruingi D (University Hospital Coventry and Warwickshire); Allen R, Al Sadek Y, Alves do Canto Brum H, Asharaf H, Ashman M, Balakumar V, Barrington J, Baskaran R, Berry A, Bhachoo H, Bilal A, Boaden L, Chia WL, Covell G, Crook D, Dadnam F, Davis L, De Berker H*, Doyle C, Fox C, Gruffydd-Davies M, Hafouda Y, Hill A, Hubbard E, Hunter A, Inpadhas V, Jamshaid M, Jandu G, Jeyanthi M, Jones T, Kantor C, Kwak SY, Malik N, Matt R, McNulty P, Miles C, Mohomed A, Myat P, Niharika J, Nixon A, O’Reilly D, Parmar K, Pengelly S, Price L, Ramsden M, Turnor R, Wales E, Waring H, Wu M, Yang T, Ye TTS, Zander A, Zeicu C (University Hospital of Wales); Bellam S, Francombe J, Kawamoto N, Rahman MR, Sathyanarayana A, Tang HT (Warwick Hospital); Cheung J, Hollingshead J, Page V, Sugarman J, Wong E* (Watford General Hospital); Chiong J, Fung E, Kan SY, Kiang J, Kok J*, Krahelski O, Liew MY, Lyell B, Sharif Z, Speake D (Western General Hospital, Edinburgh); Alim L*, Amakye NY, Chandrasekaran J, Chandratreya N, Drake J, Owoso T, Thu YM, Wellington J (Weston General Hospital); Abou El Ela Bourquin B, Alberts J, Chapman D, Rehnnuma N (West Suffolk Hospital); Ainsworth K, Carpenter H, Emmanuel T, Fisher T, Gabrel M, Guan Z, Hollows S, Hotouras A, Ip Fung Chun N, Jaffer S, Kallikas G, Kennedy N, Khan Z, Lewinsohn B, Liu FY, Mohammed S, Rutherfurd A, Situ T, Stammer A, Taylor F, Thin N, Urgesi E, Zhang N (Whipps Cross Hospital); Ahmad MA, Bishop A, Bowes A, Dixit A, Glasson R, Hatta S, Hatt K, Larcombe S*, Preece J, Riordan E, Solichan D (Worcestershire Royal Hospital); Fegredo D, Haq MZ, Li C*, McCann G, Stewart D (Wrexham Maelor Hospital); Baraza W, Bhullar D, Burt G*, Coyle J, Deans J, Devine A, Hird R, Ikotun O, Manchip G, Ross C, Storey L, Tan WWL, Tse C, Warner C, Whitehead M, Wu F (Wythenshawe Hospital); Court EL, Crisp E, Francis N, Huttman M, Mayes F, Rahman A, Robertson H*, Rosen H, Sandberg C, Smith H (Yeovil District Hospital); Al Bakry M, Ashwell W, Bajaj S, Bandyopadhyay D, Browlee O, Burway S, Chand CP, Elsayeh K*, Elsharkawi A, Evans E, Ferrin S, Fort-Schaale A, Habib S, Iacob M, I K, Impelliziere Licastro G, Mankoo AS, Olaniyan T, Otun J, Pereira R, Reddy R, Saeed D, Simmonds O, Singhal G, Tron K, Wickstone C, Williams R (York Teaching Hospital); Bradshaw E, De Kock Jewell V, Houlden C, Knight C*, Metezai H, Mirza-Davies A, Seymour Z, Spink D, Wischhusen S (Ysbyty Gwynedd, Bangor).

**Systematic review writing team**

Mwayi Kachapila, Mark Monahan, Raymond Oppong, Tracy E. Roberts, Chidubem Okeke Ogwulu.
