# Supplementary material for: Preliminary model assessing the cost-effectiveness of preoperative chlorhexidine mouthwash at reducing postoperative pneumonia among abdominal surgery patients in South Africa
Source: PLoS One. 2021 Aug 12;16(8):e0254698. doi: 10.1371/journal.pone.0254698 (PMC8360544; doi:10.1371/journal.pone.0254698)
Supplement: S1 Paper — (DOCX) [file pone.0254698.s003.docx]

**A systematic review on the costs of postoperative pneumonia following abdominal surgery**

GlobalSurg Collaborative

**Writing Committee**

Mwayi Kachapila^1,2^, Mark Monahan^1,2^, Raymond Oppong^2^, Tracy E. Roberts^1,2^, Chidubem Okeke Ogwulu^2^.

**Writing committee members’ affiliations**

1, National Institute for Health Research Global Health Research on Global Surgery Unit, Institute of Translational Medicine, University of Birmingham, Birmingham, England, United Kingdom

2, Health Economics Unit, Institute of Applied Health Research, College of Medical and Dental Sciences, University of Birmingham, Birmingham, England, United Kingdom,

**Writing committee members’ contributions**

MK chaired the writing committee, contributed to the conceptualization, collected the data, carried out the analysis, wrote the first draft and edited the manuscript. MM contributed to the conceptualization, reviewed the analysis, and edited the manuscript. RO and COO supervised the writing and edited the manuscript. TER contributed to the conceptualization, supervised writing and edited the manuscript. All authors commented on draft and approved the manuscript for publication.

Corresponding author:

Email: M.Kachapila@bham.ac.uk (MK)

**Abstract**

**Background**: Abdominal surgery is one of the most frequently performed surgeries globally. Pneumonia following abdominal surgery is associated with prolong mechanical ventilation, long length of hospital stay, high re-admission and mortality. The objective of the study was to estimate the costs associated with pneumonia following abdominal surgery aimed at informing decision makers of the amount of resources that can be saved if postoperative pneumonia incidence rates can be reduced.

**Methods**: We conducted a systematic search of literature on the costs of pneumonia following abdominal surgery. We searched Ovid EMBASE, HMIC, EconLit, Web of Science and Ovid MEDLINE(R).

**Results**: Five studies passed the inclusion criteria. The costs of patients with pneumonia were roughly double the costs of patients with no pneumonia diagnosis. The costs among patients with pneumonia ranged from $58,186 to $157,728 while the costs of patients with no pneumonia ranged from $30,040 to $95,206 and in the additional costs due to pneumonia range from $1,301 to $62,521.

**Conclusion**: Pneumonia after abdominal surgery is likely to result in catastrophic expenditures especially among the uninsured population hence the need for decision makers to put in place measures to protect people from high healthcare costs associated with postoperative pneumonia.

**Background**

It is estimated that between 28% and 32% of diseases can be treated through surgery and abdominal surgery is one of the most common surgery representing 25% of all operations (1) (2). Postoperative pneumonia prolongs length of hospital stay (LoS), increases need for mechanical ventilation and re-admission rates (3-6). Reducing postoperative pneumonia can therefore save a lot of healthcare resources. Estimating the costs of postoperative pneumonia can help decision makers to make informed decision when allocating resources to reduce postoperative pneumonia.

**Challenges in estimating the additional costs due to postoperative pneumonia**

There are methodological issues that can pose a challenge when estimating postoperative pneumonia costs. Firstly, there is no agreed period for defining postoperative pneumonia, some studies only include hospital acquired pneumonia while others include post discharge pneumonia (7). As such, there will be discrepancies in pneumonia costs between studies that are limited to hospital-acquired pneumonia and those that include post discharge pneumonia.

Secondly, the presence of confounding variables such as age and comorbidities may give a depicted impact of hospital acquired infections on the estimated cost of the infection in such scenario estimating the additional costs of an infection depends on the choice of the comparators and how best the comparators eliminate confounding (7, 8).

Lastly, a multi-hospital study by Melnick et al (9) established large variations in antimicrobial usage and resistance among the 10 hospitals implying that hospital setting affects resource use. Multi-hospital studies in this case will pool resource use and costs from a number of hospitals and are likely to be more representative than single hospital studies.

To our best knowledge, the costs of pneumonia following abdominal surgery have not been systematically searched and synthesised. This study therefore aimed at reviewing the costs of pneumonia following abdominal surgery. The findings of the study will enable clinicians and policymakers make informed decisions on healthcare interventions that can reduce postoperative pneumonia.

**Methods and materials**

**Search strategy**

[1]The searching and screening of relevant studies adopted an approach developed by Roberts et al (10). We searched Ovid EMBASE, The Health HMIC, EconLit, Web of Science and Ovid MEDLINE(R). The search strategies were organised in line with the PICO framework (11). The key search terms were abdominal surgery, pneumonia and cost. We searched the databases from their inception to April 2020 with the exception of HMIC for which was searched up to March 2020. The detailed search strategies have been included in the S1 Appendix. We also searched relevant studies from the references of the studies identified during the systematic search.

**Eligibility Criteria**

We searched for studies which had possibility of having costs of pneumonia following abdominal surgery. Studies were included if the **P**articipants had a condition in need of abdominal surgery. **I**ntervention: The intervention was abdominal surgery. **C**omparator: The comparator was absence of pneumonia at the end of the follow-up period. **O**utcome: The outcome measure was diagnosis of postoperative pneumonia after the surgery. We searched for peer reviewed studies on effectiveness studies with cost assessment, cost of illness studies or formal economic evaluations (12) published in English language.

**Exclusion criteria**

A study was excluded if:

- It was an efficacy study with no cost assessment,
- The study was not published in English language,
- The article was a study protocol, editorial, commentary, case report or conference proceeding.

**Selection of studies and data extraction**

Two reviewers (MK) and (RO) independently screened the studies found during the search, disagreements were resolved by consensus based on discussions. Data were extracted using a predefined data collection form. The following were the items extracted: authors, country of study, population, type of surgery, number of hospitals in the study, study design, cost estimation perspective, pneumonia definition, LoS, pneumonia definition and costs.

**Results**

# Summary of studies identified during literature search

# The search identified 3,160 studies of which 147 were duplicates and were deleted as shown in the PRISMA diagram Fig S1. The remaining 3,013 studies were moved to Stage I for screening. After the stage I screening, 5 studies from category A were moved to stage II. After stage II screening 5 studies were moved forward for quality appraisal (13-17). The categories of the studies have been included in S2 appendix.

Fig S1: PRISMA Flow Diagram

**Summary of the studies included in the review**

# All the 5 studies were conducted in the USA (13-17). There were differences on the types of abdominal surgery of interest across the studies: prostatectomy (16), renal transplantation (17) intra-abdominal surgery (15) and kidney transplant surgery (14). One study estimated costs across multiple surgeries and dis-aggregated the results according to surgery type (13). The number of hospitals in a study ranged from 40 (13) to 994 (15) while 3 studies indicated that they were multiple hospitals without specifying the number of hospitals (14, 16, 17). All studies queried records from general hospitals: four studies queried a national databases (13-15, 17), while one study queried database for a single state of Maryland (16). All studies used prevalence and top-down costing methodologies (18).

# Average hospital charge per patient was the economic outcome (13, 15, 16). Two studies followed patients beyond the hospitalisation period, one of the two studies followed and tracked the average patient costs up to 2 years after abdominal surgery (17) while the other study tracked costs up to three years (14). However, second and third year costs reported by Naik et al (14) were not clear and were not reported in this review. All studies reported aggregated average charges per patient of the entire follow-up period. Resource use and unit costs were not reported apart from the LoS reported in three studies (13, 15, 16). The average charges per patient were used as a proxy of costs in this review. Two studies did not report the year of the costs and we assumed the year of the costs was the last year of data collection (15, 17).

# Definition of postoperative pneumonia and confounding

# In all the 5 studies pneumonia was defined according to the International Classification of Disease version 9 (ICD-9) which defines pneumonia as the presence of pneumonia causing bacteria in the respiratory tract (19). Most studies used the pneumonia definition at the time of discharge (13, 15, 16). Pneumonia was measured as the occurrence of pneumonia at any time in the first 3 years after surgery (14) and in the first two years after surgery (17).

# Potential confounding was accounted by matching demographic variables, primary diagnosis, principle procedure, age group, urgency of admission, co-infections and discharge year (13). Pneumonia costs were estimated using regression analysis but was not stated how this accounted for potential confounding (14-17) (see Table S1).

Table S1: Data extracted

| Author | Country of study | Study population | Study Design | Pneumonia definition | Cost year | Perspective | Age group | Costs included | Direct or indirect costs | Mean costs (pneumonia) | Mean costs (Non-pneumonia) | Additional costs due to pneumonia |
| --- | --- | --- | --- | --- | --- | --- | --- | --- | --- | --- | --- | --- |
| Eber et al (13) | USA | All surgery patients, dis-aggregated by surgery type | Retrospective cohort study | Pneumonia at discharge according to ICD-9-CM | 2006 | Payer | All ages | Patient hospital charges | Direct costs | $29,922 | $17,444 | $12,479 |
| Thompson et al (15) | USA | Intra-Abdominal Surgery Patients | Cluster randomized trial | Pneumonia at discharge according to ICD-9-CM | 2006 | Payer | 18 years and older | Patient hospital charges | Direct costs | $51,642 | $20,861 | $30,780 |
| Schimtges et al (16) | USA | Prostatectomy Patients | Retrospective cohort analysis | Pneumonia at discharge according to ICD-9-CM | 2008 | Payer | NR | Patient hospital charges | Direct costs | $41,062 | $21,199 | $19,863 |
| Naik et al (14) | USA | Kidney-transplant patients | Retrospective cohort analysis | Pneumonia at discharge according to ICD-9-CM in the first year after surgery | 2011 | Payer | Not reporter | Costs associated with surgery (up to 1 year) | Direct costs | $118,967 | $72,561 | $46,406 |
| Kutinova et al (17) | USA | Renal-transplant patients | Retrospective cohort analysis | Pneumonia according to ICD-9-CM in the first 2 years after surgery | 2001 | Payer | All ages | Costs associated with surgery (up to 2 years) | Direct costs | $117,699 | $64,586 | $53,113 |

# NR = Not reported

# Costs of postoperative pneumonia

Table S2 gives the costs of abdominal surgery patients. At discharge, the average costs for patients with pneumonia ranged from $58,186 to $79,623 (15, 16) while the average costs of patients with no pneumonia ranged from $30,040 to $32,164 (15, 16). The additional costs due to pneumonia ranged from $1,301 to $62,444 (13). These results represent an increase in costs due to pneumonia between 48% and 60% (13, 15). At year one following surgery, the average costs of patients with pneumonia ranged from $118,967 to $134,923 (14, 17) while the average costs of patients with no pneumonia varied from $72,561 to $75,266 (14, 17). The resulting additional costs due to pneumonia ranged from $46,406 to $51,059 (14, 17). At two years following surgery, the average cost of patients with pneumonia was $157,728 while the average cost of patients with no pneumonia was $95,206 such that the average cost of pneumonia was $62,521 (17).

| Table S2: Pneumonia costs following abdominal surgery | | | | |
| --- | --- | --- | --- | --- |
| **Cost due to pneumonia after abdominal surgery at discharge** | | | | |
| **Author** | | **Pneumonia** | **No pneumonia** | **Additional pneumonia costs** |
| Eber et al (13) | Invasive Surgery | NR | NR | $62,444 |
|  | Elective Surgery | NR | NR | $1,301 |
| Thompson et al (15) | | $79,623 | $32,164 | $47,459 |
| Schimtges et al (16) | | $58,186* | $30,040* | *$28,146 |
| **Cost due to pneumonia after abdominal surgery (at one year)** | | | | |
| **Author** | | **Pneumonia** | **No pneumonia** | **Additional Pneumonia costs** |
| Naik et al (14) | | $118,967 | $72,561 | $46,406 |
| Kutinova et al (17) | | $134,923 | $75,266 | $51,059 |
| **Cost due to pneumonia after abdominal surgery (at 2 years)** | | | | |
| **Author** | | **Pneumonia** | **No Pneumonia** | **Additional Pneumonia costs** |
| Kutinova et al (17) | | $157,728 | $95,206 | $62,521 |

*median costs, while the rest are mean costs, NR= Not reported

**Length hospital of stay**

On average, patients who developed pneumonia spent many days in hospital (between 7 and 14.8 days) compared to patients with no pneumonia (between 3 and 6.1 days). Additional days spent in hospital due to pneumonia ranged from 4 to 20 days (see Table S3).

Table S3: Length hospital of stay (in days)

| **Study** | **Pneumonia (LoS)** | **No pneumonia (LoS)** | **Additional LoS due to pneumonia** |
| --- | --- | --- | --- |
| Eber et al (13) | 14.8 | 4.6 | 10.2 |
| Thompson et al (15) | 17.1 | 6.1 | 11 |
| Schimtges et al (16) | * 7 | *3 | *4 |
| Naik et al (14) | NR | NR | NR |
| Kutinova et al (17) | NR | NR | NR |

NR = Not reported, all days are mean days apart from days with a * which are median days

# Quality assessment results

# Methodological quality of the studies included in the synthesis were checked against the Larg and Moss checklist for cost of illness studies (20). Most studies did not report if there were uncertainties on the cost input parameters, how the uncertainties were addressed or how the assumptions made during costing could have affected the results (14-17). Most studies addressed the important cost differences between pneumonia and patients with no pneumonia (13, 15-17). Justification of the cost components and the costing methods used was made in most studies (13, 16, 17). Most studies scored just above half of the total possible score (between 10 and 12 out of 19) (14-16) while the highest score was 16 out of 19 (13). Despite the low scores, all studies measured the additional costs due to pneumonia for that reason we deemed the studies were important for this review. The scored checklist has been attached in the S3 Appendix.

**Discussion**

**Summary of the main findings**

We identified 5 studies with costs of pneumonia following abdominal surgery from which the costs in this study were estimated. The average costs of patients who had pneumonia surgery were roughly double the average costs of patients did not acquire pneumonia both at discharge and up to two years following abdominal surgery. At the same time patients who had pneumonia stayed longer in hospital compared to patients who did not acquire pneumonia. The implication is that the additional costs due to pneumonia among abdominal surgery patients are partly due to the additional LoS among patients with pneumonia.

The study has also highlighted the issues that are likely to cause disparities in pneumonia costs estimated from studies and challenges encountered in estimating the costs of pneumonia. First, the pneumonia costs increased when post discharge studies were included in the review and also increased with the follow-up period. Second, the presence of confounding variables may distort the costs of illness as such confounding must accounted either during the designing of a study, data capturing or statistical analysis stage (21). Eber et al (13) found both the lowest and highest cost differences between patients with and without pneumonia at discharge and it was the only study in which matching of the potential confounding variables was done. Due to the small number of studies in this review, it is difficult to establish the impact of matching on the costs, whether matching is associated with high or low costs. Finally, all studies were multi-hospital studies hence we did not compare the costs with single hospital studies.

**Strengths of the study**

The main strength of this study is that it is the first study to systematically search and review the cost burden of pneumonia among abdominal surgery patients. The review has presented the pneumonia costs at three different time periods: at discharge, at one and two years after the surgery.

**Limitations of the study**

This study has a number of limitations. First, all the studies included in the review were from the USA, this limitations makes the generalisability of the costs to other countries difficult. Second, the evidence presented in this study is limited to direct healthcare costs taking a payer perspective. Only direct costs related to treating the illness have been included in this review. However, costs vary according to the perspective adopted since some studies include indirect costs like lost labour hours and other costs such as transport to access services (22, 23). Evidence from studies that include wider societal costs show that indirect costs make up a large proportion of total costs (24). Hence, this review might report lower costs than the actual costs of pneumonia. Despite these limitations, based on the available evidence, the literature shown that post-operative pneumonia significantly increases the healthcare costs for abdominal surgery patients.

## Comparison with other findings

Due to unavailability of abdominal surgery studies we compared our costs findings to pneumonia costs following cardiac surgery. Thompson et al (25) found that cardiac surgery patients with postoperative pneumonia spent a day more in hospital and had higher costs at 90 days after operation compared to patients without pneumonia. Coronary artery bypass grafting surgery patients with pneumonia had $9,786 additional costs while heart valve surgery patients had $13,782 additional costs (25). Our results show that pneumonia increased average patient costs by at least 38% to utmost 60% while among cardiac surgery patients pneumonia was found to increase the costs by around 25% (25).

# Policy implications

# This study has important policy implications. All the studies included in this analysis were from the USA where the median household income in 2018 was $61,937 (26). With the additional cost per case of pneumonia ranging from $1,301 to $62,521, the pneumonia costs can be as high as the household’s annual income. Due to the very high costs associated with pneumonia, we recommend that governments should put in place reimbursement mechanisms for pneumonia especially targeting the high risk groups in out-of-pocket and private insurance settings.

# Conclusion

# This review has shown that the impact of pneumonia on healthcare resources has not been given much attention despite postoperative pneumonia having a huge cost burden. The results shown that there is a significant difference in costs between abdominal surgery patients with and with no pneumonia implying that many healthcare resources can be saved if new interventions that reduce postoperative pneumonia are explored and implemented. The high pneumonia costs are likely to have catastrophic costs as such we recommend that governments should put in place policies that will cushion the uninsured against the unplanned expenses due to pneumonia.

# References

1. Shrime MG, Bickler SW, Alkire BC, Mock C. Global burden of surgical disease: an estimation from the provider perspective. Lancet Glob Health. 2015;3 Suppl 2:S8-9.

2. McConkey SJ. Case Series of Acute Abdominal Surgery in Rural Sierra Leone. World Journal of Surgery. 2002;26(4):509-13.

3. Chughtai M, Gwam CU, Mohamed N, Khlopas A, Newman JM, Khan R, et al. The Epidemiology and Risk Factors for Postoperative Pneumonia. J Clin Med Res. 2017;9(6):466-75.

4. Kawasaki K, Yamamoto M, Suka Y, Kawasaki Y, Ito K, Koike D, et al. Development and validation of a nomogram predicting postoperative pneumonia after major abdominal surgery. Surgery today. 2019;49(9):769-77.

5. Awolaran O, Gana T, Samuel N, Oaikhinan K. Readmissions after laparoscopic cholecystectomy in a UK District General Hospital. Surgical endoscopy. 2017;31(9):3534-8.

6. Yang CK, Teng A, Lee DY, Rose K. Pulmonary complications after major abdominal surgery: National Surgical Quality Improvement Program analysis. J Surg Res. 2015;198(2):441-9.

7. Kaier K, Heister T, Götting T, Wolkewitz M, Mutters NT. Measuring the in-hospital costs of Pseudomonas aeruginosa pneumonia: methodology and results from a German teaching hospital. BMC Infectious Diseases. 2019;19(1):1028.

8. Rose S, van der Laan Mark J. Why Match? Investigating Matched Case-Control Study Designs with Causal Effect Estimation. The International Journal of Biostatistics2009.

9. Melnick G, Keeler E. The effects of multi-hospital systems on hospital prices. Journal of Health Economics. 2007;26(2):400-13.

10. Roberts T, Henderson J, Mugford M, Bricker L, Neilson J, Garcia J. Antenatal ultrasound screening for fetal abnormalities: a systematic review of studies of cost and cost effectiveness. Bjog. 2002;109(1):44-56.

11. Aslam S, Emmanuel P. Formulating a researchable question: A critical step for facilitating good clinical research. Indian J Sex Transm Dis AIDS. 2010;31(1):47-50.

12. Goodacre S, McCabe C. An introduction to economic evaluation. Emergency Medicine Journal. 2002;19(3):198-201.

13. Eber MR, Laxminarayan R, Perencevich EN, Malani A. Clinical and economic outcomes attributable to health care-associated sepsis and pneumonia. Arch Intern Med. 2010;170(4):347-53.

14. Naik AS, Dharnidharka VR, Schnitzler MA, Brennan DC, Segev DL, Axelrod D, et al. Clinical and economic consequences of first-year urinary tract infections, sepsis, and pneumonia in contemporary kidney transplantation practice. Transpl Int. 2016;29(2):241-52.

15. Thompson DA, Makary MA, Dorman T, Pronovost PJ. Clinical and economic outcomes of hospital acquired pneumonia in intra-abdominal surgery patients. Ann Surg. 2006;243(4):547-52.

16. Schmitges J, Trinh QD, Bianchi M, Sun M, Abdollah F, Ahyai SA, et al. The effect of annual surgical caseload on the rates of in-hospital pneumonia and other in-hospital outcomes after radical prostatectomy. Int Urol Nephrol. 2012;44(3):799-806.

17. Kutinova A, Woodward RS, Ricci JF, Brennan DC. The incidence and costs of sepsis and pneumonia before and after renal transplantation in the United States. Am J Transplant. 2006;6(1):129-39.

18. Luppa M, Heinrich S, Angermeyer MC, König H-H, Riedel-Heller SG. Cost-of-illness studies of depression: A systematic review. Journal of Affective Disorders. 2007;98(1):29-43.

19. US Department of Health and Human Services. Official ICD-9-CM Guidelines for Coding and Reporting. In: National Center for Health Statistics, editor. Hyattsville, MD: 2008.

20. Larg A, Moss JR. Cost-of-illness studies: a guide to critical evaluation. Pharmacoeconomics. 2011;29(8):653-71.

21. Pourhoseingholi MA, Baghestani AR, Vahedi M. How to control confounding effects by statistical analysis. Gastroenterol Hepatol Bed Bench. 2012;5(2):79-83.

22. Teni FS, Gebresillassie BM, Birru EM, Belachew SA, Tefera YG, Wubishet BL, et al. Costs incurred by outpatients at a university hospital in northwestern Ethiopia: a cross-sectional study. BMC health services research. 2018;18(1):842.

23. Rice DP. Estimating the cost of illness. Am J Public Health Nations Health. 1967;57(3):424-40.

24. Chen A, Gupte C, Akhtar K, Smith P, Cobb J. The Global Economic Cost of Osteoarthritis: How the UK Compares. Arthritis. 2012;2012:698709.

25. Thompson MP, Cabrera L, Strobel RJ, Harrington SD, Zhang M, Wu X, et al. Association Between Postoperative Pneumonia and 90-Day Episode Payments and Outcomes Among Medicare Beneficiaries Undergoing Cardiac Surgery. Circ Cardiovasc Qual Outcomes. 2018;11(9):e004818.

26. Bureau USC. Household Income: 2018, American Community Survey Briefs. <https://www.census.gov/content/dam/Census/library/publications/2019/acs/acsbr18-01.pdf>: Household Income: 2018, American Community Survey Briefs; 2018.

# S1 Appendix: Database search Strategies

1. **EconLit**

abdominal surgery AND pneumonia OR respiratory tract infection AND cost (7)

1. **Web of science**

TOPIC: (abdominal surgery) AND TITLE: (pneumonia or Respiratory Tract Infections) AND TITLE: (cost) (86)

1. **Database: HMIC Health Management Information Consortium** <1979 to March 2020> Search Strategy:

1 exp Abdominal surgery/ or exp Surgery/ (5791)

2 exp Surgery/ (5791)

3 exp Laparotomy/ (26)

4 1 or 2 or 3 (5791)

5 exp Pneumonia/ (224)

6 exp Staphylococcal pneumonia/ (1)

7 exp Staphylococcal pneumonia/ (1)

8 pneumonia.mp. (596)

9 (pneumonia or bronchopneumonia).ti,mp. (600)

10 exp Respiratory Tract Infections/ (570)

11 (((chest adj2 infection?) or thorax) adj2 infection?).mp,ti,ab. (23)

12 exp adverse events/ (753)

13 ((((advers ad2 event? or adverse) adj2 outcome?) or negative) adj2 outcome?).ti,mp,ab. (791)

14 5 or 6 or 7 or 8 or 9 or 10 or 11 or 12 or 13 (2660)

15 exp Costs/ (7084)

16 exp Hospital costs/ (80)

17 exp Treatment costs/ (409)

18 exp Unit costs/ (119)

19 cost.mp. (19693)

20 cost.mp. (19693)

21 exp Economics/ (5860)

22 Economics/ or exp Economics, Hospital/ (597)

23 exp Economics, Medical/ or Economics/ (597)

24 exp Economics, Pharmaceutical/ or Economics.mp. (5349)

25 (absenteeism or presenteeism).mp. (519)

26 (((Sick adj2 leave) or sick) adj2 day?).mp. (51)

27 (catastrophic adj2 cost?).mp. (12)

28 ((((disability adj2 day?) or work) adj2 day?) or workday?).mp. (301)

29 budget$.mp. (6217)

30 (spend or spending).mp. (3759)

31 (((((((labo?r adj2 market) or labo?r) adj2 supply) or labo?r) adj2 income) or labo?r) adj1 force).mp. (274)

32 (((((indirect adj2 cost?) or economic) adj2 cost?) or economic) adj2 burden).mp. (196)

33 (employment or unemployment or productivity or earning? or wage?).mp. (14436)

34 (((((cost adj2 burden) or direct) adj2 cost?) or direct) adj2 expenditure?).mp. (9)

35 15 or 16 or 17 or 18 or 19 or 20 or 21 or 22 or 23 or 24 or 25 or 26 or 27 or 28 or 29 or 30 or 31 or 32 or 33 or 34 (49310)

36 4 and 14 and 35 (7)

**Database: Ovid MEDLINE(R) <1946 to April Week 3 2020> Search Strategy:**

1 exp General Surgery/ (38589)

2 surgery.ti,ab,kw. (973879)

3 (general adj3 surgery).ti,ab,kw. (15409)

4 (surgical adj3 procedure?).ti,ab,kw. (88379)

5 1 or 2 or 3 or 4 (1043882)

6 exp Pneumonia/ (91640)

7 exp Pneumonia, Pneumocystis/ or Pneumonia, Pneumococcal/ (13653)

8 (pneumonia or bronchopneumonia).ti,ab,kw. (103390)

9 exp Respiratory Tract Infections/ (353991)

10 (((chest adj2 infection?) or thorax) adj2 infection?).ti,ab,kw. (1417)

11 exp Adverse Outcome Pathways/ (80)

12 exp "Drug-Related Side Effects and Adverse Reactions"/ (114606)

13 ((((advers ad2 event? or adverse) adj2 outcome?) or negative) adj2 outcome?).ti,ab,kw. (51729)

14 (post adj1 operative adj1 complication?).ti,ab,kw. (5490)

15 6 or 7 or 8 or 9 or 10 or 11 or 12 or 13 or 14 (565164)

16 "Costs and Cost Analysis"/ (48386)

17 exp Economics/ (595916)

18 Economics/ or exp Economics, Hospital/ (51358)

19 exp Economics, Medical/ or Economics/ (41129)

20 exp Economics, Pharmaceutical/ or Economics/ (30088)

21 (absenteeism or presenteeism).ti,ab,kw. (5453)

22 (((Sick adj2 leave) or sick) adj2 day?).ti,ab,kw. (1059)

23 (catastrophic adj2 cost?).ti,ab,kw. (118)

24 ((((disability adj2 day?) or work) adj2 day?) or workday?).ti,ab,kw. (6011)

25 budget$.ti,kw,ab. (23411)

26 (spend or spending).ti,kw,ab. (22807)

27 (((((((labo?r adj2 market) or labo?r) adj2 supply) or labo?r) adj2 income) or labo?r) adj1 force).ti,ab,kw. (3417)

28 (((((indirect adj2 cost?) or economic) adj2 cost?) or economic) adj2 burden).ti,ab,kw. (8172)

29 (employment or unemployment or productivity or earning? or wage?).ti,ab,kw. (107338)

30 ((workforce cost? or work) adj1 force adj1 cost?).ti,ab,kw. (3)

31 (((((cost adj2 burden) or direct) adj2 cost?) or direct) adj2 expenditure?).ti,ab,kw. (287)

32 16 or 17 or 18 or 19 or 20 or 21 or 22 or 23 or 24 or 25 or 26 or 27 or 28 or 29 or 30 or 31 (721659)

33 5 and 15 and 32 (733)

**Database: Embase <1974 to 2020 April 23> Search Strategy:**

1 exp General Surgery/ (15025)

2 (general adj3 surgery).ti,ab,kw. (25836)

3 (surgical adj3 procedure?).ti,ab,kw. (147605)

4 1 or 2 or 3 (178119)

5 exp Pneumonia/ (291520)

6 exp Pneumonia, Pneumocystis/ or Pneumonia, Pneumococcal/ (17302)

7 (pneumonia or bronchopneumonia).ti,ab,kw. (170336)

8 (((postoperative adj2 pneumonia) or postoperative) adj2 period).ti,ab,kw. (45861)

9 exp chest infection/ (3053)

10 exp respiratory tract infection/ (387971)

11 exp postoperative complication/ (658238)

12 (((chest adj2 infection?) or thorax) adj2 infection?).ti,ab,kw. (2925)

13 exp adverse event/ (611680)

14 exp adverse outcome/ (48328)

15 exp adverse outcome pathway/ (344)

16 ((((advers ad2 event? or adverse) adj2 outcome?) or negative) adj2 outcome?).ti,ab,kw. (95627)

17 5 or 6 or 7 or 8 or 9 or 10 or 11 or 12 or 13 or 14 or 15 or 16 (1894899)

18 exp Cost?/ and Cost Analysis/ (21856)

19 exp "cost utility analysis"/ (9566)

20 exp "cost effectiveness analysis"/ (149732)

21 exp "cost of illness"/ (19041)

22 economics/ (237218)

23 exp economics hospital/ (836740)

24 exp economics medical/ (836740)

25 exp economics pharmaceutical/ (200812)

26 (absenteeism or presenteeism).ti,ab,kw. (8972)

27 (catastrophic adj2 cost?).ti,ab,kw. (176)

28 (((Sick adj2 leave) or sick) adj2 day?).ti,ab,kw. (1655)

29 ((((disability adj2 day?) or work) adj2 day?) or workday?).ti,ab,kw. (9643)

30 budget$.ti,kw,ab. (38680)

31 ((workforce cost? or work) adj1 force adj1 cost?).ti,ab,kw. (4)

32 (((((cost adj2 burden) or direct) adj2 cost?) or direct) adj2 expenditure?).ti,ab,kw. (503)

33 (employment or unemployment or productivity or earning? or wage?).ti,ab,kw. (158851)

34 18 or 19 or 20 or 21 or 22 or 23 or 24 or 25 or 26 or 27 or 28 or 29 or 30 or 31 or 32 or 33 (1139039)

35 4 and 17 and 34 (2320)

36 exp General Surgery/ (15025)

37 (general adj3 surgery).ti,ab,kw. (25836)

38 (surgical adj3 procedure?).ti,ab,kw. (147605)

39 36 or 37 or 38 (178119)

40 exp Pneumonia/ (291520)

41 exp Pneumonia, Pneumocystis/ or Pneumonia, Pneumococcal/ (17302)

42 (pneumonia or bronchopneumonia).ti,ab,kw. (170336)

43 (((postoperative adj2 pneumonia) or postoperative) adj2 period).ti,ab,kw. (45861)

44 exp chest infection/ (3053)

45 exp respiratory tract infection/ (387971)

46 exp postoperative complication/ (658238)

47 (((chest adj2 infection?) or thorax) adj2 infection?).ti,ab,kw. (2925)

48 exp adverse event/ (611680)

49 exp adverse outcome/ (48328)

50 exp adverse outcome pathway/ (344)

51 ((((advers ad2 event? or adverse) adj2 outcome?) or negative) adj2 outcome?).ti,ab,kw. (95627)

52 40 or 41 or 42 or 43 or 44 or 45 or 46 or 47 or 48 or 49 or 50 or 51 (1894899)

53 exp Cost?/ and Cost Analysis/ (21856)

54 exp "cost utility analysis"/ (9566)

55 exp "cost effectiveness analysis"/ (149732)

56 exp "cost of illness"/ (19041)

57 economics/ (237218)

58 exp economics hospital/ (836740)

59 exp economics medical/ (836740)

60 exp economics pharmaceutical/ (200812)

61 (absenteeism or presenteeism).ti,ab,kw. (8972)

62 (catastrophic adj2 cost?).ti,ab,kw. (176)

63 (((Sick adj2 leave) or sick) adj2 day?).ti,ab,kw. (1655)

64 ((((disability adj2 day?) or work) adj2 day?) or workday?).ti,ab,kw. (9643)

65 budget$.ti,kw,ab. (38680)

66 ((workforce cost? or work) adj1 force adj1 cost?).ti,ab,kw. (4)

67 (((((cost adj2 burden) or direct) adj2 cost?) or direct) adj2 expenditure?).ti,ab,kw. (503)

68 (employment or unemployment or productivity or earning? or wage?).ti,ab,kw. (158851)

69 53 or 54 or 55 or 56 or 57 or 58 or 59 or 60 or 61 or 62 or 63 or 64 or 65 or 66 or 67 or 68 (1139039)

70 39 and 52 and 69 (2320)

# S2 Appendix: Category of the studies screened

# Stage I categories

Below is the criteria for determining the relevance of each study after screening the title and abstract. The number of studies in each category have been included in brackets.

1. The study reports on costs of pneumonia following abdominal surgery (5),
2. The study presents an economic evaluation of abdominal surgery related to pneumonia (0),
3. The study may have useful information but does not fall into categories A or B (16),
4. The study presents an economic evaluation of abdominal surgery not related to pneumonia (98);
5. The study reports costs of pneumonia but not in relation to abdominal surgery (79),
6. The study reports respiratory complications following abdominal surgery but not costs (15),
7. The study discusses the adverse effects of surgery (996),
8. The study does not have any relevance to economic evaluation nor has cost information related to pneumonia following abdominal surgery (1,804).

# Stage II

Full texts of studies moved forward to Stage II were screened against the eligibility criteria. After the screening, the Studies were further categorised into other 6 categories:

1. The study reports the additional economic costs of pneumonia following abdominal surgery (5),
2. The study reports economic costs of pneumonia but not related to abdominal surgery (0),
3. The study reports abdominal surgery costs but not related to pneumonia (0),
4. The study does not present any cost information on either pneumonia or abdominal surgery (0),
5. The study presents the adverse effects of abdominal surgery (0),
6. The article was a conference summary, conference abstract, protocol, commentary editorial or was not published in English language (0).

**S3 Appendix: The Larg and Moss checklist for cost-of-illness studies**

| No | **Checklist criterion** | **Author** | | | | |
| --- | --- | --- | --- | --- | --- | --- |
|  | **Theme 1: Analytical framework** | **Eber et al (13)** | **Kutinova et al (17)** | **Thompson et al (15)** | **Schimtges et al (16)** | **Naik et al (14)** |
| 1 | Was the appropriate epidemiologic approach taken? | Y | Y | Y | Y | Y |
| 2 | Was the study question well specified? | Y | Y | Y | Y | Y |
|  |  |  |  |  |  |  |
|  | **Theme 2: Methodology and data** |  |  |  |  |  |
| 3 | additional, or excess, costs were measured? | Y | Y | Y | Y | Y |
| 4 | only costs specific to (caused by) the health problem were included (confounders controlled)? | Y | Y | Y | Y | Y |
| 5 | all important effects were captured? | Y | Y | Y | Y | CT |
| 6 | important differences across subpopulations were accounted for? | Y | Y | Y | Y | Y |
| 7 | the required level of detail could be provided? | Y | Y | Y | N | Y |
| 8 | Was the resource quantification method(s) well executed? | Y | Y | Y | Y | N |
| 9 | Were healthcare resources valued appropriately? | Y | Y | Y | Y | CT |
| 10 | Was the approach for valuing production losses justified, and assumptions valid? | CT | N | N | N | N |
| 11 | Was the inclusion of intangible costs appropriate: | CT | N | N | N | N |
|  | **Theme 3: analysis and reporting** |  |  |  |  |  |
| 12 | Did the analysis address the study question? | Y | Y | Y | Y | Y |
| 13 | Was a range of estimates presented? | Y | N | Y | Y | Y |
| 14 | Were the main uncertainties identified? | Y | N | N | N | N |
| 15 | Was a sensitivity analysis performed? | Y | N | N | N | N |
| 16 | Was adequate documentation and justification given for cost components, data and sources, assumptions and methods? | Y | Y | Y | N | N |
| 17 | Was uncertainty around the estimates and its implications adequately discussed? | N | N | N | N | N |
| 18 | Were important limitations discussed regarding the cost components, data, assumptions and methods? | Y | Y | N | N | Y |
| 19 | Were the results presented at the appropriate level of detail to answer the study question (cost components; disease subtypes, severity, stage; subpopulation groups, cost bearers)? | Y | Y | N | N | Y |
|  | **Final Score** | 16 | 13 | 12 | 10 | 10 |
| *CT= Cannot tell, Y = yes ,N =No | | |  |  |  |  |
