# Supplementary figures and images for: Preliminary model assessing the cost-effectiveness of preoperative chlorhexidine mouthwash at reducing postoperative pneumonia among abdominal surgery patients in South Africa
Source: PLoS One. 2021 Aug 12;16(8):e0254698. doi: 10.1371/journal.pone.0254698 (PMC8360544; doi:10.1371/journal.pone.0254698)

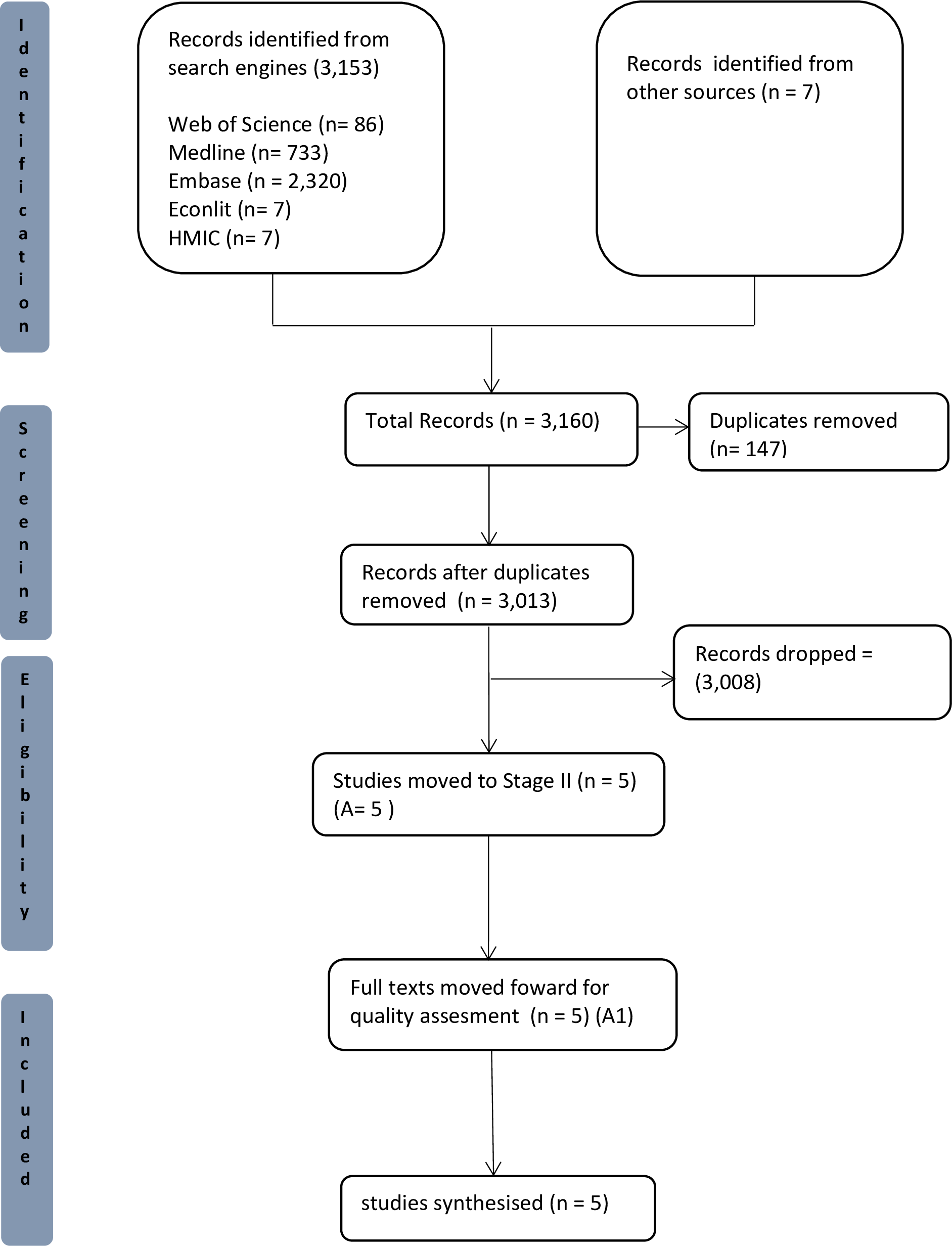

Supplement: S1 Fig — (TIF) [file pone.0254698.s004.tif]
